# PKM2 inhibitor suppresses kidney fibrogenesis by disrupting YAP-TEAD-CCN2 transcriptional signaling following ischemia–reperfusion injury

**DOI:** 10.1016/j.jbc.2025.111029

**Published:** 2025-12-09

**Authors:** Wakako Kosakai, Tsutomu Inoue, Tetsuya Sato, Hirokazu Okada

**Affiliations:** 1 Department of Nephrology, Faculty of Medicine, Saitama Medical University, Saitama, Japan; 2 Biomedical Research Center, Faculty of Medicine, Saitama Medical University, Saitama, Japan

**Keywords:** β-catenin, CCN2, compound 3k, epithelial cell, metabolic reprogramming, PKM2, proximal tubular epithelial cell, renal fibrosis, TEAD, YAP1

## Abstract

Fibrosis progressively impairs organ function and drives the progression of chronic kidney disease (CKD), for which effective targeted therapies are lacking. Although metabolic reprogramming toward glycolysis promotes fibrosis, the molecular link between metabolic shifts and transcriptional control in CKD and its therapeutic potential has not yet been established. In this study, we demonstrate that pyruvate kinase M2 (PKM2) orchestrates renal fibrosis *via* nuclear translocation and interaction with the Yes-associated protein (YAP) and beta-catenin (β-catenin) transcriptional networks. Using unilateral ischemia–reperfusion injury mouse models and human renal tubular epithelial cells, we revealed that pharmacological inhibition and genetic knockdown of PKM2 markedly attenuate renal atrophy and the expression of fibrotic markers, including cellular communication network factor 2. Mechanistically, compound 3k inhibited the nuclear translocation of PKM2 and YAP, thereby suppressing TEA domain transcription factor (TEAD)-mediated communication network factor 2 transcription. Similarly, siRNA-mediated silencing of *PKM2* further confirmed the inhibition of YAP-TEAD signaling. Furthermore, co-immunoprecipitation confirmed that PKM2 forms complexes with YAP and β-catenin, integrating metabolic and transcriptional regulation. Our findings provide direct evidence that PKM2 promotes fibrosis in CKD through its role as a transcriptional cofactor rather than *via* its enzymatic activity. Notably, PKM2 inhibition by compound 3k remained effective even with delayed intervention, suggesting clinical translatability. Overall, these findings highlight PKM2 as a key integrator of metabolic and transcriptional reprogramming in kidney fibrosis and provide crucial preclinical evidence supporting PKM2-targeted strategies in CKD.

Fibrosis represents a leading cause of organ failure worldwide, accounting for up to 45% of deaths in industrialized countries (1). In the context of the kidneys, this condition is known as chronic kidney disease (CKD) (2, 3). Despite advances in understanding CKD pathogenesis, the lack of effective therapies to prevent or reverse CKD progression remains a critical unmet medical need (4).

Current therapeutic approaches, including renin-angiotensin system inhibitors, such as angiotensin-converting enzyme inhibitors, angiotensin receptor blockers, and sodium-glucose cotransporter 2 inhibitors,

provide modest renoprotection and fail to address the fundamental metabolic and transcriptional dysregulation driving fibrotic progression. CKD typically develops following acute kidney injury, and damaged tubular epithelial cells play a central role in its progression (5, 6, 7). During the chronic phase, hypoxia resulting from hypoperfusion and ischemia induces metabolic reprogramming, contributing to CKD progression (8). Furthermore, hypoxia contributes to chronic inflammation and fibrosis by altering cellular metabolism, including glucose metabolism (9). This metabolic shift toward glycolysis provides energy for cellular repair processes and leads to the generation of biosynthetic precursors that support fibroblast proliferation and extracellular matrix production.

The role of pyruvate kinase M2 (PKM2), an alternatively spliced isoform of *PKM*, in mediating these pathological metabolic changes has garnered considerable attention (10, 11, 12). *PKM* encodes two distinct isoforms, M1 and M2, generated as PKM1 and PKM2 through the insertion of exons 9 and 10 during mRNA splicing, respectively (10, 13, 14). PKM1 and PKM2 differ by 22 amino acids and have distinct functionality owing to this discrepancy (10). Unlike the constitutively active tetrameric PKM1, PKM2 exists in both tetrameric and dimeric forms, enabling it to have dual functions in metabolism and signaling (15, 16). Depending on energy supply, tetrameric PKM2 promotes glycolysis and energy production, whereas dimeric PKM2 reduces glycolytic flux and redirects intermediates towards anabolic pathways. Notably, dimeric PKM2 also exhibits protein kinase activity, modulating gene expression and signal transduction (15, 17).

The Yes-associated protein (YAP) and TEA domain transcription factor (TEAD) signaling pathway has emerged as a critical regulator of renal fibrosis progression. YAP is a key transcriptional coactivator in the Hippo signaling pathway (18), and its activation promotes the expression of profibrotic genes, such as communication network factor 2 (*CCN2*) (formerly known as *CTGF*), through its interaction with TEAD (19). The YAP1–TEAD axis promotes cell proliferation, epithelial-mesenchymal transition (EMT), and extracellular matrix production, thereby contributing to renal fibrosis. This pathway also integrates mechanical cues with metabolic signaling, indirectly regulating glycolysis-associated gene expression and supporting aerobic glycolysis and anabolic metabolism to sustain cell proliferation (15, 19, 20).

Although β-catenin contributes to tissue repair following acute kidney injury (21), it also promotes EMT, extracellular matrix production, and profibrotic factor expression, ultimately leading to CKD progression (22). Additionally, dimeric PKM2 translocates to the nucleus and regulates β-catenin-mediated transcriptional activity (21), highlighting PKM2 and β-catenin as compelling targets for elucidating the crosstalk between metabolic regulation and transcriptional control in various pathological conditions.

The role of PKM2 modulation has been explored in diabetic kidney disease (DKD) using TEPP46 and shikonin, which exert opposing effects. TEPP46 serves as an activator by promoting PKM2 tetramer formation and enhancing its enzymatic activity, whereas shikonin acts as an inhibitor by disrupting PKM2 activity (23, 24, 25, 26). However, the mechanisms underlying their kidney-protective effects remain unclear. Moreover, the role of PKM2 modulation in non-DKDs remains unclear (27). sodium-glucose cotransporter 2 inhibitors protect against DKD by suppressing excessive glucose influx into the proximal tubular epithelial cells, which possibly involves PKM2 (28, 29) given its role in abnormal glucose metabolism.

Furthermore, the antifibrotic effects of compound 3k, a PKM2-selective allosteric inhibitor derived from a novel naphthoquinone, have not been established in CKD models beyond DKD. Notably, although extensive evidence implicates PKM2, YAP/TEAD, and β-catenin in renal metabolic reprogramming and fibrosis, the precise mechanisms through which PKM2 nuclear translocation coordinates with transcriptional coactivator networks to drive CKD progression remain unknown (23, 30, 31). Moreover, whether selective PKM2 inhibition can uncouple metabolic and transcriptional drivers of fibrosis *in vivo* has not been directly explored.

Therefore, in this study, we aimed to elucidate the molecular mechanisms linking PKM2 nuclear localization to fibrogenic transcription (17, 32) and assess the therapeutic efficacy of compound 3k in attenuating renal fibrosis using a unilateral ischemia–reperfusion injury (UIRI) mouse model (33). By integrating cell-specific genetic labeling, pharmacological and genetic PKM2 suppression, and advanced protein interaction analyses, this study reveals new insights into the interplay between metabolic regulation and transcriptional control in CKD and provides a basis for targeted antifibrotic therapy. Overall, this approach addresses a critical gap in the literature regarding the link between cellular metabolism and transcriptional regulation in kidney fibrosis and provides novel preclinical evidence for a therapeutic strategy that directly targets PKM2-dependent coactivator networks.

## Results

### Compound 3k attenuates renal fibrosis in the UIRI mouse model

**Figure 1:**
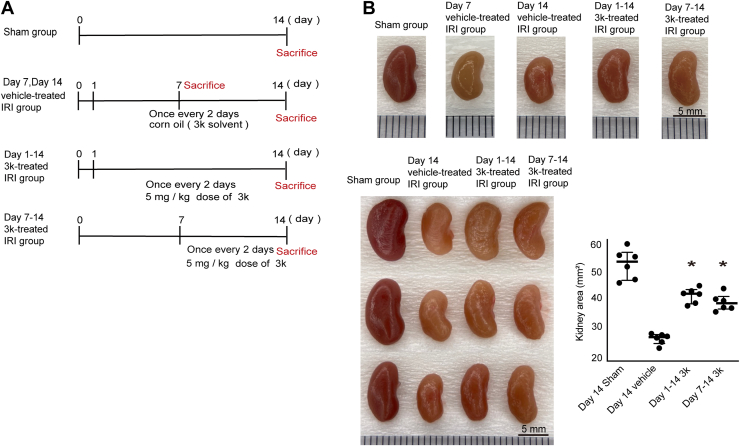
***In vivo* analysis of the anti-fibrotic effects of compound 3k in a mouse unilateral ischemia–reperfusion injury (UIRI) model**. *A*, experimental protocol for the mouse UIRI model, including treatment regimens and time points for sacrifice in the sham, vehicle-treated, and 3k-treated groups. *B*, representative images of mouse kidneys 7 and 14 days after UIRI under various experimental conditions: sham, vehicle-treated (corn oil), and 3k-treated groups. The kidney area was quantitatively measured and presented as the median with IQR. Statistical analysis was performed using Steel’s test for group comparisons *versus* the vehicle group, with statistical significance defined as *p* < 0.05. Individual data points represent the median and IQR (N = 6). The detailed statistical methods are described in the Statistical Analysis section of the Experimental procedures. (In *panel B*, the kidneys in the upper row are the same specimens as those in the lower panel (Day 14 series) of this progressive fibrosis model, and the images are reused to highlight representative kidneys from each treatment group at Day 14 and to illustrate that renal atrophy is more advanced at Day 14 than at Day 7.). UIRI, unilateral ischemia–reperfusion injury.

Six-week-old male *C57BL/6* mice were randomly divided into five groups: sham-operated mouse group [sham group], vehicle-treated mice with UIRI [day 7 and day 14 vehicle-treated IRI groups], early intervention group receiving compound 3k (5 mg/kg BW) starting 1 day after UIRI surgery [day 1–14 compound 3k-treated IRI group], and a delayed intervention group receiving the same dose of compound 3k starting 7 days after UIRI [day 7–14 compound 3k-treated IRI group] (Fig. 1*A*). Macroscopic examination of kidneys harvested seven or 14 days after surgery revealed marked renal atrophy in the positive control groups compared with that in the negative control group. Conversely, renal atrophy development was effectively suppressed in the early- and delayed-compound 3k intervention groups (Fig. 1*B*). Quantification of kidney long-axis sectional area (median [interquartile range, IQR], mm^2^) on day 14, measured from scale-calibrated images using ImageJ, revealed the following: sham 53.4 [49.3–55.2], vehicle 32.0 [30.9–32.5], day 1 to 14 compound 3k 44.4 [42.3–45.0], and day 7 to 14 compound 3k 41.4 [40.3–42.7] (N = 6 per group).

Statistical analysis was performed using Steel’s test, with the Day 14 vehicle-treated group used as the control. Both day 1 to 14 and day 7 to 14 compound 3k treatment groups exhibited significantly greater kidney areas compared with the vehicle group (∗*p* < 0.05 vs. vehicle; Steel’s test). Error bars represent the IQR (Fig. 1*B*).

### Compound 3k suppresses fibrotic gene expression in mouse kidneys

**Figure 2:**
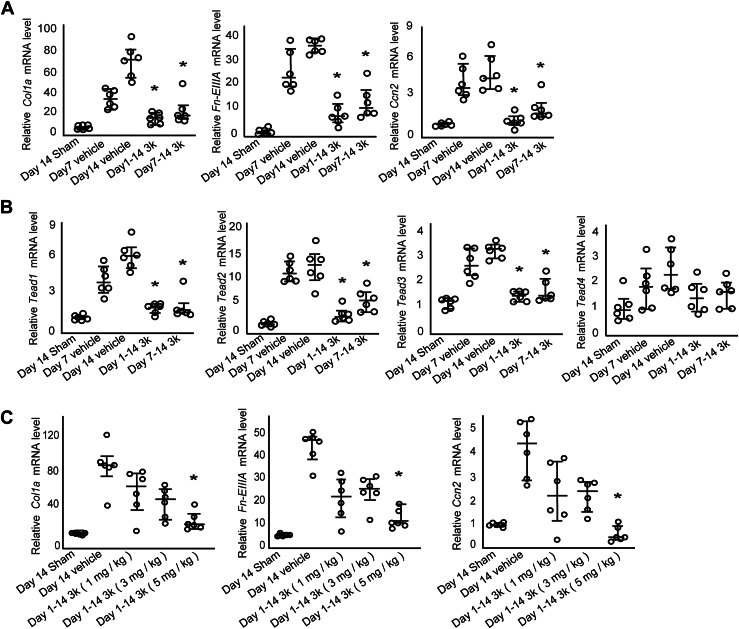
***In vivo* quantitative reverse transcription PCR analysis of the anti-fibrotic effects of compound 3k in a mouse UIRI model**. *A*, quantitative reverse transcription PCR analysis of total RNA extracted from whole kidney tissues 7 and 14 days after UIRI. The genes analyzed include collagen type 1α, fibronectin EIIIA, cellular communication network factor 2 (*Ccn2*), and (*B*) TEA domain transcription factors 1 *to* 4 (*Tead1–4*). *C*, dose-dependent effects of 3k treatment. The therapeutic effect of compound 3k (1, 3, and 5 mg/kg) was evaluated by measuring the mRNA levels of extracellular matrix components (Collagen type Iα and Fibronectin EIIIA isoform) and *Ccn2* in mouse kidneys. The results were analyzed using the Steel test. ∗indicates statistical significance (*p* < 0.05) compared with the vehicle-treated group at 14 days (Day 14 vehicle). Individual data points represent the median and IQR (N = 6). UIRI, unilateral ischemia–reperfusion injury.

Quantitative reverse transcription-PCR (RT-qPCR) analysis of total RNA isolated from kidneys on day 14 post-surgery revealed a notable upregulation in the expression of genes encoding extracellular matrix components that accumulate during kidney disease progression, including collagen type Iα, fibronectin EIIIA isoform, and the profibrotic factor Ccn2, in all groups (Fig. 2*A*). However, both early and delayed interventions with compound 3k significantly suppressed their expression. Similarly, all four *Tead* family members, the primary transcriptional regulators of *Ccn2*, exhibited expression patterns similar to those of the extracellular matrix components and *Ccn2* (Fig. 2*B*). Therefore, based on extracellular matrix and *Ccn2* expression levels, these findings indicate that the therapeutic effects of compound 3k demonstrated clear dose-dependency (Fig. 2*C*).

### Compound 3k attenuates renal fibrosis and nuclear PKM2 accumulation in proximal tubules

**Figure 3:**
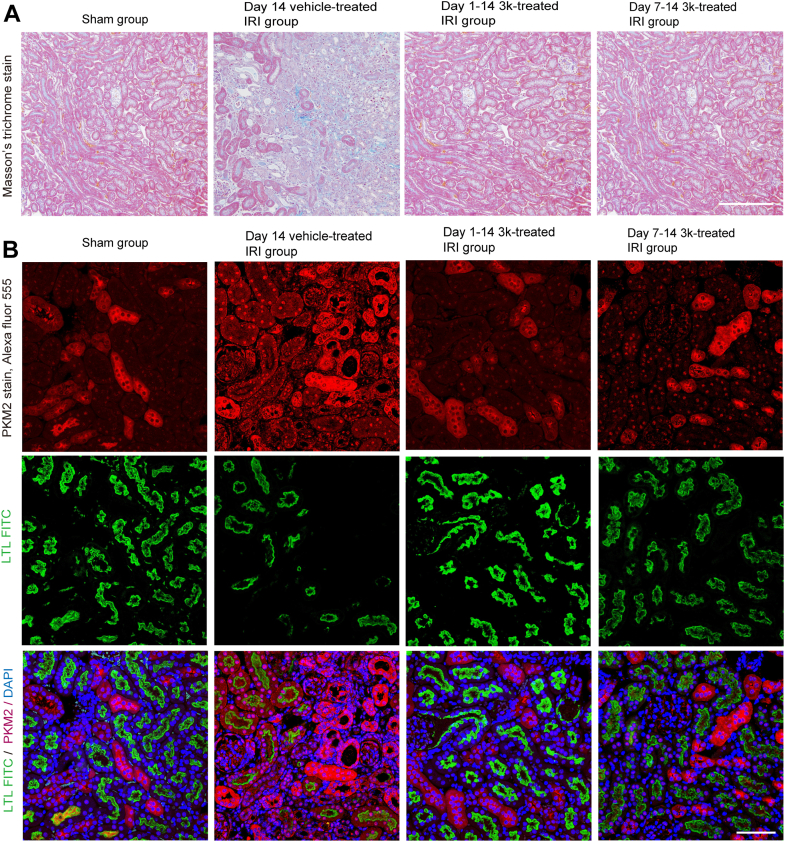
**Evaluation of renal fibrosis using Masson's trichrome and PKM2 immunofluorescence staining**. *A*, representative images of kidney sections stained with Masson’s trichrome 14 days following UIRI. This scale bar represents 500 μm. *B*, didney sections stained with anti-PKM2 antibody (Alexa Fluor 555: *red*) and *Lotus tetragonolobus* lectin (LTL; FITC: *green*). The cell nuclei were counterstained with DAPI (*blue*) (N = 6). This scale bar represents: 100 μm. Data were acquired using a confocal laser scanning microscope. LTL, Lotus tetragonolobus lectin; UIRI, unilateral ischemia–reperfusion injury.

Masson's trichrome staining revealed marked progression of fibrosis and tubular atrophy in the vehicle-treated group compared with that in the sham group (Fig. 3*A*). In contrast, both compound 3k-treated groups exhibited minimal fibrosis progression, with significantly lower fibrotic area compared to that in the sham group. Immunofluorescence staining with anti-PKM2 antibody and *Lotus tetragonolobus* lectin (LTL), a proximal tubule brush border marker, revealed a limited number of PKM2-positive tubules in the sham group (Fig. 3*B*). A representative higher-magnification image illustrating the co-localization of PKM2 and LTL in proximal tubular cells is presented in Figure S1. Conversely, the vehicle-treated group exhibited decreased LTL staining, indicating proximal tubular injury, along with widespread PKM2 positivity in tubules, including proximal tubules, with some exhibiting staining patterns indicative of nuclear accumulation. In the compound 3k-treated groups, the nuclear density and staining intensity of PKM2 were significantly decreased, whereas the number of LTL-stained cells was increased compared with those in the vehicle-treated group.

To validate the cell type-specific localization and mechanism underlying nuclear PKM2 accumulation, we employed γ-glutamyl transpeptidase promoter-driven Cre (γGT-Cre) × tandem dimer Tomato (tdTomato) reporter mice subjected to UIRI (14 days), as previously described (34). In this model, the γGT promoter drives Cre recombinase expression in cortical tubular epithelial cells, resulting in tdTomato fluorescence exclusively in these cells following recombination. Immunofluorescence analysis (Fig. S2) revealed that nuclear PKM2 staining was strongly and specifically induced in tdTomato-positive cortical tubular epithelial cells following injury (30, 34, 35).

**Figure 4:**
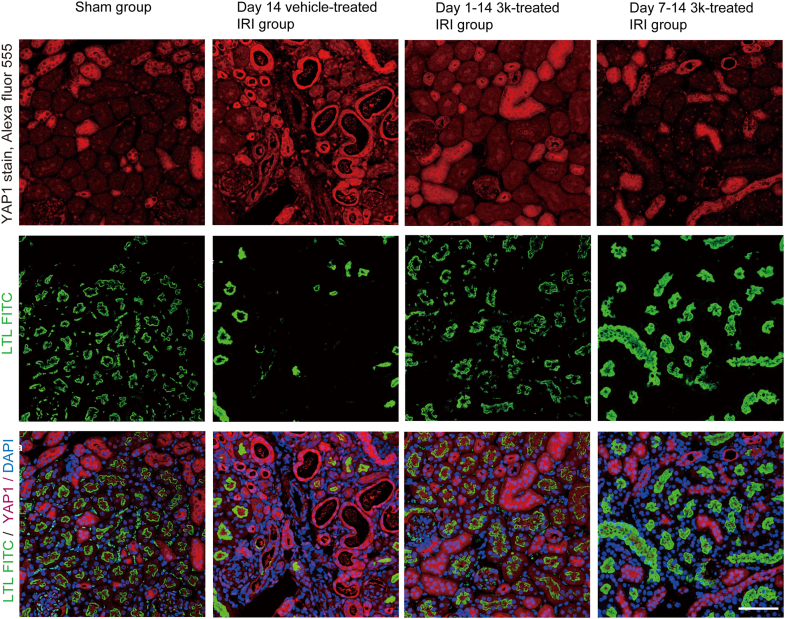
**Localization of Yap1 identified using fluorescent immunofluorescence staining**. Immunofluorescence staining of mouse proximal tubular epithelial cells highlighting YAP1 localization in relation to tubular injury markers. YAP1 was detected using an Alexa Fluor 555-conjugated antibody (*red*), and the proximal tubular marker LTL was labeled with FITC (*green*). The cell nuclei were counterstained with DAPI (*blue*) (N = 6). This scale bar represents 100 μm. Data were acquired using a confocal laser scanning microscope. LTL, Lotus tetragonolobus lectin.

Subsequently, we performed immunofluorescence staining for YAP1, a known oncogene that plays a key role in cell proliferation, glycolysis, and survival (36) (Fig. 4). It was overexpressed in proximal tubular epithelial cells following IRI: in the sham group, YAP1 staining was limited to a few tubules, whereas in the vehicle-treated group, positive staining was observed in both the cytoplasm and some nuclei of dilated tubules. Both early and delayed intervention with compound 3k reduced YAP1 staining to levels comparable to those in the sham group.

**Figure 5:**
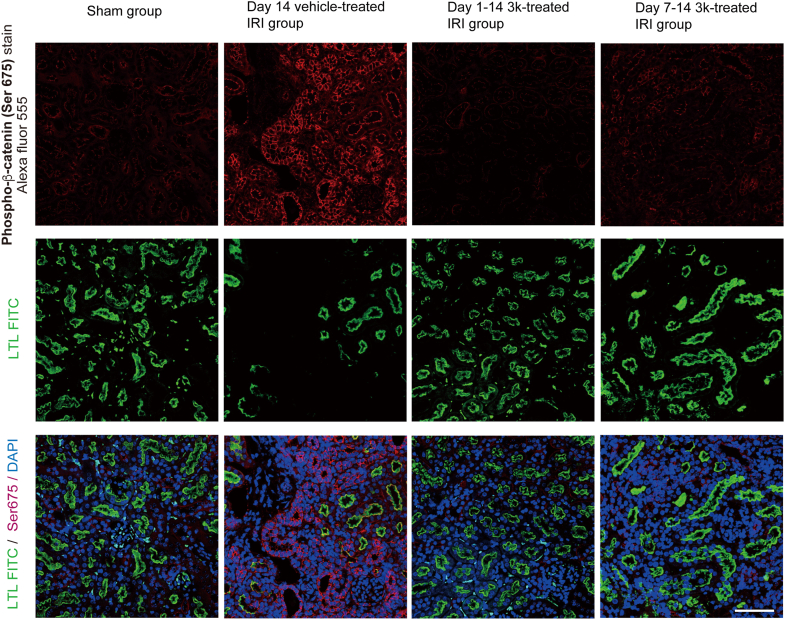
**Immunofluorescence staining of phospho-β-catenin (p-β-catenin, Ser 675) in mouse kidneys was performed to determine the state of phosphorus metabolism**. Immunofluorescence staining of p-β-catenin at Ser675 in mouse renal tissue sections. p-β-catenin was detected using an Alexa Fluor 555-conjugated antibody (*red*); the proximal tubular marker LTL was labeled with FITC (*green*), and nuclei were counterstained with DAPI (*blue*) (N = 6). This scale bar represents 100 μm. Data were acquired using a confocal laser scanning microscope. LTL, Lotus tetragonolobus lectin.

**Figure 6:**
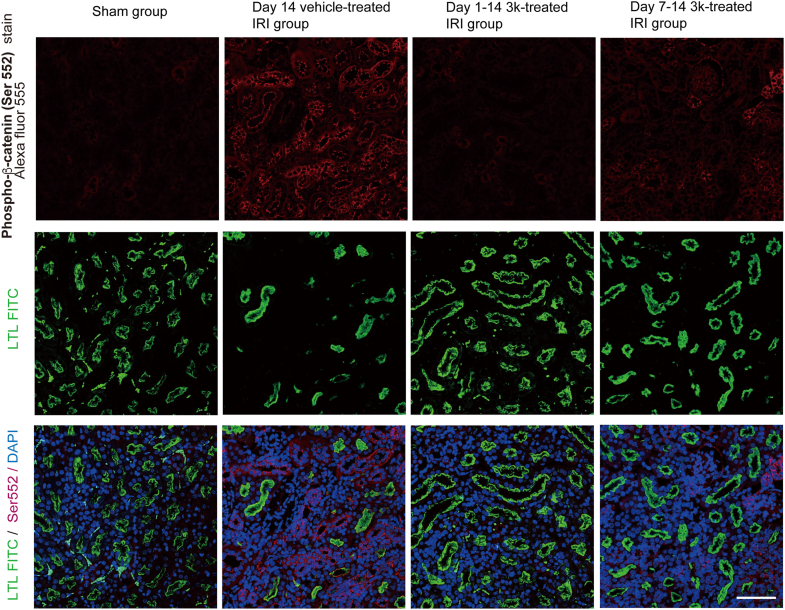
**Immunofluorescence staining of p-β-catenin (Ser552) in mouse kidneys was performed to determine the state of phosphorus metabolism**. Immunofluorescence staining of p-β-catenin at Ser552 in mouse renal tissue sections. p-β-catenin was detected using an Alexa Fluor 555-conjugated antibody (*red*); the proximal tubular marker LTL was labeled with FITC (*green*), and nuclei were counterstained with DAPI (*blue*) (N = 6). This scale bar represents 100 μm. Data were acquired using a confocal laser scanning microscope. LTL, Lotus tetragonolobus lectin.

Next, we performed immunofluorescence staining for β-catenin phosphorylated at Ser675 and Ser552 (Figs. 5 and 6). Phosphorylation at these sites enhances the co-transactivator function of β-catenin by promoting its nuclear translocation and binding to T-cell factor/lymphoid enhancer factor (37). Immunofluorescence analysis revealed strong expression of β-catenin phosphorylated at Ser675 and Ser552 in UIRI kidneys, whereas only weak expression was observed in the sham and compound 3k-treated groups. These findings suggest that both Ser675 and Ser552 phosphorylation and transcriptional activation occur during renal injury. Positive staining for phosphorylated β-catenin (p-β-catenin) was weaker in both early and delayed intervention groups than in the vehicle-treated group.

### Compound 3k preserves HK-2 cell viability at experimental concentrations

Cell viability was assessed using Cell Counting Kit-8 (CCK-8; Dojindo) according to the manufacturer’s instructions. The results revealed that treatment with compound 3k at two and 4 μM had no significant effect on the survival rate of HK-2 cells, which maintained a viability of approximately 100% (Fig. S3, *A* and *B*). Accordingly, these concentrations were selected for subsequent experiments to ensure effective PKM2 inhibition without cytotoxicity. HK-2 cells treated with the same concentrations of dimethyl sulfoxide (DMSO; vehicle control), which was used as a solvent for compound 3k, showed no change in viability.

### Compound 3k suppresses fibrotic gene expression in OGD/R-injured HK-2 cells

**Figure 7:**
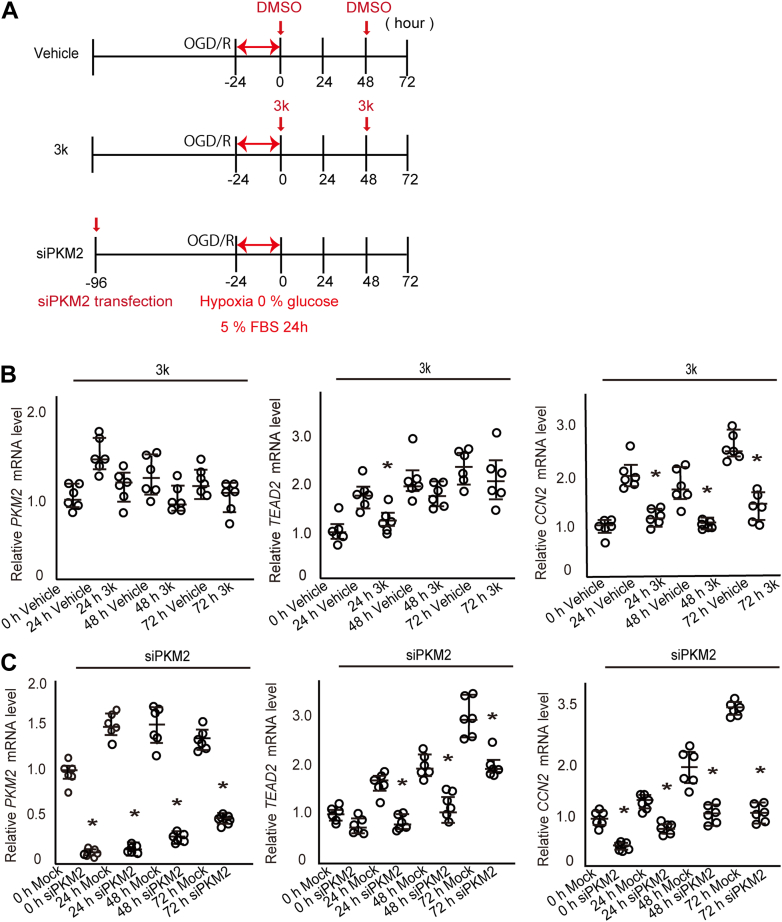
***In vitro* analysis of 3k treatment and siPKM2 transfection in a human renal tubular epithelial (HK-2) cell injury model**. *A*, time course analysis of the effects of oxygen–glucose deprivation and reoxygenation (OGD/R) and 3k treatment. mRNA expression analysis of *PKM2*, *TEAD2*, and *CCN2* in (*B*) 3k-treated and (*C*) siPKM2-transfected cells, from 24 to 72 h. Statistical comparisons between the control or the mock and treatment groups at each time point were performed using the Wilcoxon test. ∗ indicates statistical significance (*p* < 0.05). Individual data points represent the median and IQR (N = 6).

To further validate the above findings, we performed experiments using an *in vitro* ischemia–reperfusion model using HK-2 cells (oxygen–glucose deprivation and reoxygenation: OGD/R (36)), which serves as a model for CCN2 induction (Fig. 7*A*). This model mimics the metabolic stress and hypoxic conditions observed in the UIRI mouse model. We examined *PKM2*, *CCN2*, and *TEAD*2 mRNA expression levels from 24 to 72 h using RT-qPCR (Fig. 7*B*). Although *PKM2* expression exhibited an increasing trend up to 24 h, compound 3k treatment did not significantly alter *PKM2* expression levels at any time point. Moreover, compound 3k significantly decreased *TEAD2* expression at 24 h, although no notable changes in expression were observed up to 72 h. In contrast, *CCN2* expression was consistently and significantly suppressed in the compound 3k-treated group from 24 to 72 h. These results indicate that pharmacological inhibition of PKM2 directly modulates *CCN2* and *TEAD2* expression levels, suggesting a potential regulatory relationship between these factors.

To further explore this mechanism, we evaluated another enzymatic PKM2 inhibitor, compound 7d (38), which inhibits PKM2 metabolic activity by binding to ATP/ADP. Under the same experimental conditions, including a 48 h *in vitro* OGD/R protocol, treatment with compound 7d did not decrease CCN2 expression (Fig. S5). In contrast, under the same conditions, compound 10i (39), an FBP allosteric site inhibitor structurally similar to compound 3k, reduced CCN2 expression in a dose-dependent manner (33, 40). Collectively, these results indicate that both compound 3k and compound 10i suppress CCN2 expression, but not compound 7d, in the *in vitro* OGD/R model.

**Figure 8:**
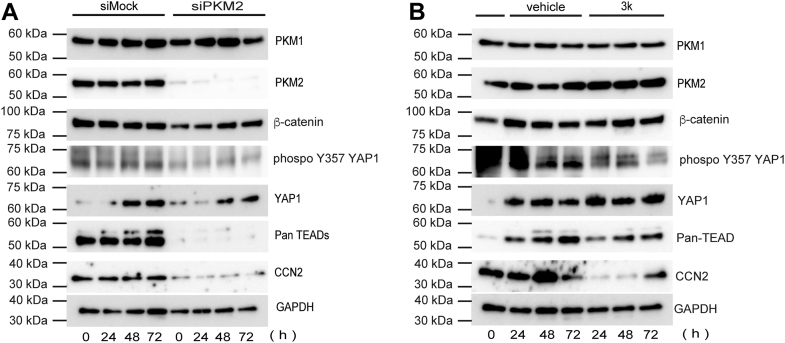
**Western blot analysis of protein expression in 3k-treated and siPKM2-transfected HK-2 cells**. *A*, western blot analysis of siPKM2-transfected HK-2 cells. *B*, western blot analysis of 3k-treated HK-2 cells.

Next, we transfected HK-2 cells with siRNA targeting *PKM2* and induced CCN2 expression using the OGD/R protocol (Fig. 7*C*). In Figure 8*A*, the 0 h time point represents the start of hypoxia/reoxygenation following 72 h of siRNA treatment (as shown in Fig. 7), at which point PKM2 knockdown had already been achieved.

*PKM2* expression was significantly suppressed at 0 h and consistently remained suppressed for up to 72 h, even after induction of *PKM2* expression in the mock siRNA-transfected group following reoxygenation at 24 h. The time course of *CCN2* expression closely mirrored that of *TEAD2* expression, with the expression of both genes consistently and significantly suppressed in the *PKM2* siRNA-transfected group compared with that in the mock transfection group from 0 to 72 h. RT-qPCR analysis of *TEAD* family transcription factors (*TEAD1–4*) following siRNA-mediated *PKM2* knockdown in OGD/R-injured HK-2 cells (Fig. S4, *A*–*D*, N = 3) revealed that only *TEAD2* mRNA expression was selectively and significantly reduced by *PKM2* knockdown. Conversely, no significant changes in the expression levels of *TEAD1*, *TEAD3*, and *TEAD4* were observed under these conditions.

Western blot analysis of proteins extracted from siPKM2-transfected HK-2 cells (Fig. 8*A*) revealed that the expression of PKM2, β-catenin, YAP1, Pan-TEADs, and CCN2 significantly decreased between 24 and 72 h post-transfection. In contrast, PKM1 expression did not change compared with that in the control, confirming the specificity of siRNA targeting. These results demonstrate that knocking down *PKM2* in HK-2 cells using siRNA significantly decreased the expression of its target proteins. Similarly, treatment with compound 3k (Fig. 8*B*) did not significantly alter PKM2 expression levels within the same time frame, consistent with its mechanism as a functional inhibitor rather than a transcriptional suppressor.

The significant reduction in Pan-TEAD and CCN2 expression following compound 3k treatment suggests that this compound has a targeted or selective regulatory effect on these proteins. Consistent with the RT-qPCR results, compound 3k treatment did not affect PKM2 protein levels, whereas *PKM2* siRNA transfection effectively knocked down *PKM2* expression. Furthermore, the TEAD protein levels changed in parallel with CCN2 protein levels across all time points. Notably, neither compound 3k treatment nor *PKM2* siRNA transfection affected PKM1 protein levels. Furthermore, compound 3k treatment did not affect total β-catenin or YAP1 protein levels compared with those in the vehicle-treated group. However, both treatments reduced the levels of the dephosphorylated form of YAP1 (Tyr357), which is associated with nuclear translocation (41). In contrast, siPKM2 transfection, but not compound 3k treatment, also reduced total β-catenin and YAP1 protein levels compared to the mock transfection.

To further examine isoform specificity, we performed additional western blotting analyses using a TEAD2-specific antibody (Fig. S7). Both compound 3k treatment and siPKM2 knockdown consistently reduced TEAD2 protein levels, suggesting that PKM2-dependent regulation of TEAD is primarily mediated *via* the TEAD2 isoform in this disease context.

Additionally, we examined the effects of transforming growth factor beta (TGF-β) stimulation and compound 3k treatment on canonical and non-canonical profibrotic pathways at the protein level using Western blot analyses (Fig. S6). TGF-β1 stimulation markedly increased phosphorylated Smad3 and CCN2 protein levels (42, 43, 44), whereas TEAD2 protein expression was not changed significantly. Treatment with compound 3k, either alone or combined with TGF-β1, reduced TEAD2 and CCN2 protein levels (33, 45), whereas phosphorylated Smad3 levels remained elevated across all TGF-β1-stimulated conditions.

### Compound 3k and PKM2 knockdown suppress the nuclear accumulation of YAP1 and β-catenin

**Figure 9:**
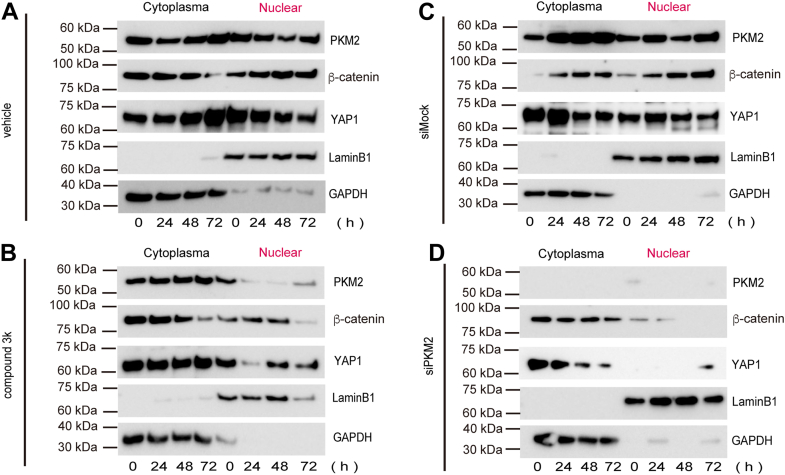
**Western blot analysis of cell fraction protein expression in 3k-treated and siPKM2-transfected HK-2 cells**. *A* and *B*, Western blot analysis of cytoplasmic and nuclear protein fractions from 3k-treated HK-2 cells to determine β-catenin and YAP1 nuclear translocation. *C* and *D*, western blot analysis of cytoplasmic and nuclear protein fractions from siPKM2-transfected HK-2 cells to determine β-catenin and YAP1 nuclear translocation (N = 6).

To further examine the nuclear translocation of β-catenin and YAP1, whose total protein levels remained unchanged following compound 3k treatment or *PKM2* siRNA transfection, we prepared cytoplasmic and nuclear protein fractions from HK-2 cells (Fig. 9, *A*–*D*). GAPDH and lamin B1 were used as markers for cytoplasmic and nuclear fractions, respectively, and confirmed appropriate compartmental localization. Comparison between the compound 3k-treated and vehicle-treated groups revealed no substantial differences in the β-catenin, YAP1, and PKM2 protein levels in the cytoplasmic fraction, consistent with the whole cell lysate Western blot results. However, analysis of the nuclear fraction showed that the proportion of nuclear PKM2 was consistently decreased in the 3k-treated group compared with that in the vehicle-treated group across all time points. Similar trends were observed for β-catenin and YAP1, with 3k treatment suppressing their nuclear translocation (Fig. 9*B*).

A comparison between the *PKM2*-siRNA and mock transfection groups confirmed that siRNA transfection effectively knocked down *PKM2*, consistent with the Western blot analysis of the non-fractionated protein samples. Although differences in the cytoplasmic fractions between the *PKM2*-siRNA and mock transfection groups were less pronounced, analysis of the nuclear fraction revealed a marked reduction in the nuclear translocation of β-catenin and YAP1 (Fig. 9*D*). In control cells, PKM2, YAP1, and β-catenin were highly expressed in both the cytoplasm and the nucleus. However, in compound 3k-treated cells, PKM2 was highly expressed in the cytoplasm, whereas its expression in the nucleus was relatively weak. Similarly, the nuclear expression of YAP1 and β-catenin proteins was decreased following compound 3k treatment. YAP1 plays a key role in tissue reprogramming during cell proliferation and promotes glycolysis through reoxygenation, and knocking down *YAP1* reportedly reduces PKM2 expression (46).

### Compound 3k increases monomeric PKM2 and suppresses PKM2 oligomerization

**Figure 10:**
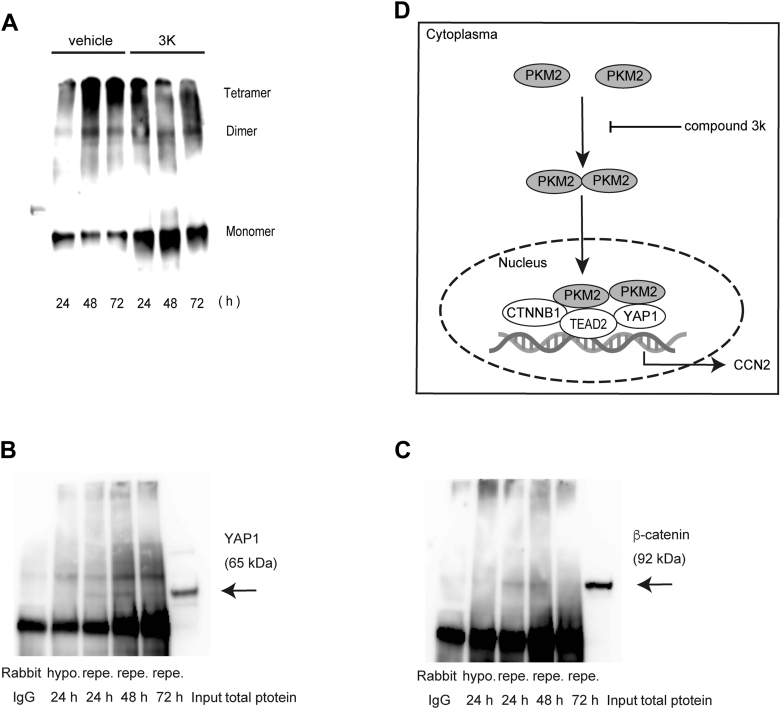
**Western blot analysis of cross-linked protein expression and Co-IP analysis in HK-2 cells**. *A*, western blot analysis of cross-linked proteins extracted from 3k-treated HK-2 cells. *B* and *C*, Co-IP analysis of the interaction between YAP1, β-catenin, and PKM2 in HK-2 cells. IP was performed using protein samples from HK-2 cells overexpressing CCN2. Co-IP with an anti-PKM2 antibody confirmed that both β-catenin and YAP1 co-precipitated with PKM2 (N = 6), (*D*) as illustrated in the schematic diagram. CCN2, cellular communication network factor 2.

To examine changes in PKM2 oligomerization induced by compound 3k treatment, we performed Western blot analysis on cross-linked proteins extracted from compound 3k-treated HK-2 cells (Fig. 10*A*). The compound 3k-treated group exhibited a higher proportion of monomeric PKM2 than the vehicle-treated control group across all time points, confirming that compound 3k treatment effectively suppressed PKM2 oligomer formation. The increase in monomeric PKM2 expression, accompanied by a decrease in tetrameric and dimeric protein expression, indicates that 3k treatment disrupts the stability or assembly of PKM2 protein complexes.

### PKM2 forms a complex with β-catenin and YAP1 in HK-2 cells

To investigate potential complex formation between PKM2 and β-catenin or YAP1, we performed immunoprecipitation analyses. Immunoprecipitated protein samples were collected from the DMSO-treated control group, which served as a vehicle control, where elevated CCN2 levels were detected (Fig. 10, *B* and *C*). Co-IP analysis using an anti-PKM2 antibody revealed that both β-catenin and YAP1 co-precipitated with PKM2, indicating that PKM2 interacts with β-catenin and YAP1, forming a complex. This interaction was confirmed through reciprocal IP assays, confirming the specificity of these observed protein interactions.

## Discussion

In this study, we demonstrated that PKM2 serves as a cofactor in the regulation of CCN2 expression and may contribute to the progression of renal fibrosis by regulating YAP-TEAD and CCN2 expression in proximal tubular epithelial cells by forming transcriptional complexes of YAP1 and β-catenin. Furthermore, compound 3k treatment ameliorated interstitial fibrosis and tubular atrophy in our progressive mouse kidney injury model by suppressing PKM2 oligomerization in tubular epithelial cells, thereby reducing CCN2 expression *via* the YAP-TEAD and β-catenin-mediated pathways.

Interstitial fibrosis, which is more strongly associated with renal dysfunction than glomerular damage (5, 6, 7), represents a promising therapeutic target in CKD. Among the mediators of fibrosis, CCN2 plays a crucial role. It has previously been demonstrated that suppressing CCN2 expression in tubular epithelial cells can mitigate renal fibrosis after UIRI in a mouse model (5, 6, 7). Furthermore, YAP1, a known oncogene, is overexpressed in proximal tubular epithelial cells and plays an important role in cell proliferation, glycolysis, and survival (36). YAP1 expression is reportedly upregulated in injured renal proximal tubular epithelial cells and localized to the nucleus (36, 47). Furthermore, knocking down *YAP1* significantly downregulates PKM2 expression and suppresses glucose uptake and lactate production (46).

Following UIRI in the kidney, some injured proximal tubular epithelial cells undergo cell death, whereas the remaining surviving cells attempt to recover from the injury and initiate a regenerative response. YAP1 is activated during this process, and its localization dynamics play an essential role. During the early stages, YAP1 is primarily localized to the cytoplasm, where it is involved in cell survival and proliferation under the regulation of the Hippo pathway. Therefore, cytoplasmic YAP1 is considered a part of the regenerative response following injury. However, when tissue stress caused by ischemia persists or the repair process fails, YAP1 translocates to the nucleus and binds to the transcription factor TEAD, inducing the expression of pro-fibrotic factors. In this study, YAP1 staining was limited to a small number of tubules in the sham group (Fig. 4). This is consistent with previous studies showing that YAP1 expression was upregulated in injured renal proximal tubular epithelial cells and localized to the nucleus (36, 47).

Under inactive conditions, YAP1 is typically localized to the cytoplasm but translocates to the nucleus upon activation, where it functions as a transcriptional regulator. In this study, Tyr357 expression was reduced in both siPKM2-transfected and compound 3k-treated HK-2 cells (Fig. 8, *A* and *B*). As Tyr357 dephosphorylation is essential for activating downstream target genes associated with fibrosis progression and YAP1 nuclear accumulation, these results suggest that interventions inhibiting PKM2 expression may suppress YAP1-mediated transcriptional responses and ameliorate fibrosis progression. The formation of the PKM2–β-catenin–YAP1 complex suggests that PKM2 may facilitate or stabilize the nuclear translocation of YAP1, thereby influencing its transcriptional activity. However, further investigation is necessary to elucidate whether the formation of this complex directly contributes to YAP1-mediated gene regulation and clarify its functional consequences in the nuclear compartment. The clinical relevance of our findings is highlighted by emerging evidence that metabolic reprogramming toward glycolysis is a hallmark of CKD progression across various etiologies. Therefore, modulating this pathway through PKM2 inhibition represents a paradigm shift from treating individual symptoms to addressing the fundamental metabolic dysregulation underlying fibrosis. The therapeutic window demonstrated by both early and delayed intervention protocols suggests clinical feasibility for patients across various stages of kidney injury.

To precisely elucidate the cell-type specificity of nuclear PKM2 induction, we employed a genetic labeling strategy using γGT-Cre × tdTomato reporter mice (Fig. S2) (48, 49). This approach enabled reliable identification of cortical tubular epithelial cells even in severely damaged tissue, overcoming the limitations of LTL staining that arise after injury (50, 51). These findings demonstrate that epithelial nuclear PKM2 is the primary inducible factor in this model, with induction observed specifically in epithelial cells and not in interstitial fibroblasts or other cell types. Representative images and immunofluorescence data from the γGT-Cre × tdTomato reporter mice confirm that nuclear PKM2 staining was strongly and specifically induced in tdTomato-positive cortical tubular epithelial cells following injury (Fig. S2).

Consistent with these observations, in the day 14 sham group, PKM2 expression was predominantly detected in the cytoplasm of kidney tubular epithelial cells, whereas in the day 14 vehicle-treated IRI group, PKM2 was strongly expressed in the nucleus. Conversely, nuclear PKM2 expression was markedly reduced in compound 3k-treated IRI groups. Furthermore, immunofluorescence staining of YAP1 and p-β-catenin at Ser675 and Ser552 confirmed their increased nuclear localization in injured tissue (Figs. 5 and 6) (52). Taken together, these results suggest that phosphorylation at these sites facilitates the nuclear accumulation and enhanced transcriptional activity of β-catenin (37), establishing a mechanistic link between PKM2 nuclear translocation, YAP/β-catenin signaling, and renal fibrosis progression (53, 54).

Comparative analysis between the *PKM2* siRNA-transfected and mock transfection groups confirmed the effective knockdown of *PKM2* following siRNA transfection, consistent with the Western blot results for the non-fractionated protein samples. Although the differences in the cytoplasmic fraction between the groups were subtle, analysis of the nuclear fraction revealed a significant decrease in the nuclear translocation of β-catenin and YAP1 following *PKM2* knockdown (Fig. 9*D*). The consistent reduction in PKM2 and CCN2 expression in siPKM2-transfected cells across all time points highlights the crucial role of PKM2 in maintaining their expression, suggesting its role in transcriptional or post-transcriptional regulation.

To determine the selectivity of compound 3k, we evaluated PKM1 protein expression levels following treatment. Our results demonstrated that compound 3K did not alter PKM1 expression, whereas it markedly suppressed PKM2-associated signaling (Fig. 8*B*). Given that PKM1 forms a constitutive tetramer and lacks the regulatory allosteric pocket targeted by compound 3k in PKM2, these findings suggest that the observed effects of compound 3k in this study are unlikely to be mediated by PKM1 inhibition. This is further supported by the known structural incompatibility of PKM1 with allosteric inhibition by compound 3k, despite its modest reported IC_50_ selectivity ratio.

To investigate the potential complex formation between PKM2 and β-catenin or YAP1, we performed IP analysis on samples from the vehicle-treated IRI group, which exhibited elevated CCN2 protein levels (Fig. 10, *B* and *C*). Co-IP analysis using anti-PKM2 antibodies confirmed that both β-catenin and YAP1 co-precipitated with PKM2, indicating that PKM2 forms a complex with β-catenin and YAP1. Therefore, the interaction between β-catenin and YAP1, mediated by PKM2, is crucial for their function within the nucleus. Inactive YAP1 is typically localized to the cytoplasm; however, upon activation, it translocates to the nucleus, where it functions as a transcriptional regulator. Furthermore, the presence of a nuclear PKM2 dimer suggests that it has a transcriptional regulatory function distinct from its role in glycolysis (55).

These findings suggest that PKM2 and YAP1 exhibit distinct subcellular localization patterns under different pathological conditions. For instance, the cytoplasmic expression of PKM2 and YAP1 observed in the day 14 sham group reflects a basal distribution, whereas their nuclear translocation in the day 14 vehicle-treated IRI group suggests their role in cellular stress response or metabolic reprogramming under pathological conditions. In particular, the decrease in nuclear PKM2 and YAP1 expression in the compound 3k-treated IRI group suggests that 3k treatment may attenuate the pathological nuclear translocation of PKM2 and YAP1, potentially by modulating cellular stress- or proliferation-associated pathways. This observation indicates a potential mechanism through which compound 3k treatment may attenuate CKD progression by regulating PKM2 localization and function (56). Moreover, our findings indicate that the interaction between β-catenin and YAP1, mediated by PKM2, is crucial for their nuclear function.

As illustrated in the schematic diagram in Figure 10*D*, PKM2 dimers form a transcriptional complex with β-catenin, YAP1, and TEAD2 in the nucleus of proximal tubule epithelial cells to regulate CCN2 expression (55). Collectively, these results suggest that PKM2 regulates TEAD2 expression and may contribute to renal fibrosis progression by modulating CCN2 expression in tubular epithelial cells through the formation of transcriptional complexes with YAP1 and β-catenin. Furthermore, compound 3k treatment may have ameliorated interstitial fibrosis and tubular atrophy in our progressive mouse kidney injury model by suppressing PKM2 oligomerization in tubular epithelial cells, thereby reducing CCN2 expression through YAP1-TEAD and β-catenin-mediated pathways.

Previous research has shown that coordinated signaling among the TGF-β, YAP/transcriptional co-activator with PDZ-binding motif (TAZ)-TEAD, and wingless-type MMTV integration site family pathways is a key determinant of organ fibrosis. Furthermore, non-canonical TEAD programs can drive CCN2 expression independently of TGF-β (54, 57). Recent studies also implicate PKM2 as a signaling hub in fibrogenesis that can reinforce canonical and noncanonical transcriptional responses (58).

Our findings further demonstrate that, although TGF-β1 stimulation strongly induced CCN2 expression and Smad3 phosphorylation, consistent with canonical EMT signaling, the TEAD2 pathway exerted a stronger regulatory influence on CCN2 in our model. Specifically, pharmacological or genetic inhibition of PKM2 effectively reduced CCN2 and TEAD2 expression independently of TGF-β1/Smad3 activation, whereas TEAD2 levels were only modestly affected by TGF-β1 alone.

This suggests that in renal tubular epithelial cells under metabolic or hypoxic stress, the PKM2–TEAD2 axis functions as a dominant regulator of CCN2 transcription, potentially overriding or acting in parallel with canonical TGF-β1 signaling (54, 57).

Collectively, these data indicate the existence of a non-canonical, TEAD-mediated transcriptional program regulating CCN2 inducibility. This program may contribute to the resistance against traditional anti-TGF-β therapies in fibrotic kidney disease and highlights the PKM2–TEAD2 circuit as a therapeutic target distinct from EMT signaling.

Several compounds have been demonstrated to regulate PKM2 polymerization. For instance, TEPP-46 activates PKM2 by stabilizing its tetrameric form (59), whereas compound 3k antagonizes PKM2 by disrupting its tetrameric structure and inducing monomer formation (60). Previous studies have reported contradictory results regarding the mechanisms through which various compounds regulate PKM2 activity in DKD models. For example, the PKM2 activator TEPP-46 ameliorates fibrosis by promoting and stabilizing tetramer formation. It also reduces HIF-1α accumulation and suppresses EMT and abnormal glucose metabolism (61). Research on PKM2 function in renal fibrosis has also revealed that PKM2 dimers play a key pathological role, with TEPP-46 exerting a protective effect in a renal fibrosis model by reducing glycolysis (61). However, these findings are based on studies centered on diabetic nephropathy. To the best of our knowledge, the present study is the first to focus on the PKM2 dimer transcription factor complex in nondiabetic nephropathy.

In contrast to the above findings, shikonin, a PKM2 inhibitor, suppresses fibrosis progression by inhibiting oligomer formation (23, 30). Despite their contrasting effects on PKM2 activity, both TEPP-46 and shikonin may inhibit PKM2 dimer formation. Our results suggest that modulation of PKM2 activity is a promising therapeutic strategy even in nondiabetic CKD. However, this study has certain limitations. First, the possible involvement of PKM2 in fibroblast function cannot be completely excluded. Further research using diverse cell type-specific analyses and experimental approaches, such as selective inhibition or gene modulation, is needed to clarify the role of PKM2 in fibroblast biology and fibrosis progression. Second, although we demonstrated complex formation between PKM2 or YAP1 and β-catenin through Co-IP analysis, direct binding has not yet been verified. Further research is warranted to translate this elucidated PKM2-YAP1-TEAD2 and β-catenin interaction into clinical application. Third, although compound 3k showed promising efficacy, dose–response relationships and pharmacokinetic–pharmacodynamic modeling in the context of kidney disease require further investigation. Fourth, although the present study provides cell type-specific evidence for a decrease in nuclear PKM2 within tubular epithelial cells following compound 3k treatment, we did not directly assess PKM2 enzymatic activity or substrate utilization *in vivo*. Further research incorporating genetically labeled tubular cell isolation and direct biochemical assays is needed to quantify PKM2 enzyme activity and clarify its metabolic function in renal fibrosis. Finally, clinical translation will require validation in human kidney disease models and assessment of potential off-target effects in other organ systems. Nevertheless, although direct binding relationships require further validation, our findings provide strong evidence for the therapeutic potential of PKM2 inhibition in preventing CKD progression.

## Experimental procedures

### Sex as a biological variable

Male mice were employed based on known sex-related differences in renal fibrosis (62), as female mice are less suitable as a fibrosis model owing to the antifibrotic effects of estrogen. Thus, male mice were employed to ensure consistency and reproducibility. Currently, it remains unclear whether the results are generalizable to female mice, representing an important area for further investigation.

### Animals

Six-week-old male *C57BL/6* mice (20–25 g) were purchased from CLEA Japan. The mice were housed under specific pathogen-free conditions at the Saitama Medical University. The environment was maintained at an optimal temperature (23 °C ± 3 °C) and humidity (50% ± 10%) with a 12:12 h light/dark cycle. Additionally, F1 mice on a C57BL/6J background were generated by crossing γGT-Cre mice with B6.Cg-Gt(ROSA)26Sortm14(CAG-tdTomato) Hze/J (Ai14, JAX#007914). In the resulting offspring, Cre-loxP–mediated excision of a STOP cassette in the Ai14 allele enabled specific and permanent tdTomato fluorescence in renal proximal tubular epithelial cells.

### UIRI model development

Briefly, the body temperature of the mice was monitored using a rectal thermometer and maintained at 37 °C using an adjustable heating pad. Anesthesia was induced with 4%–5% isoflurane and maintained with 2%–3% isoflurane throughout the procedure. Ischemia–reperfusion injury was induced in the left kidney using a unilateral lateral abdominal approach, with 18 min of ischemia to establish a progressive UIRI model (6).

### Pharmacological inhibition experiments

To investigate the role of PKM2 in the progression of acute kidney injury to CKD, the mice were randomly divided into five groups (n = 6 per group).

(a) Sham group (negative control): Mice underwent a sham operation without UIRI surgery. Only the skin and fascia were incised to control for the effects of the surgical procedure.
(b) Day 7 vehicle-treated IRI group: Mice underwent UIRI surgery and received vehicle treatment (solvent only; 10 μl of DMSO + 90 μl of corn oil) starting from post-operative day 1, administered every other day. These mice were sacrificed on day 7.
(c) Day 14 vehicle-treated IRI group: Mice underwent UIRI surgery and received vehicle treatment (solvent only) starting from post-operative day 1, administered every other day. These mice were sacrificed on day 14.
(d) Day 1 to 14 compound 3k-treated IRI group: Mice underwent UIRI surgery and received compound 3k treatment (5 mg/kg BW) starting from post-operative day 1, administered every other day. These mice were sacrificed on either day 7 or 14 to assess the effects of early intervention.
(e) Day 7 to 14 compound 3k-treated IRI group: Mice underwent UIRI surgery and received compound 3k treatment (5 mg/kg BW) starting from post-operative day 7, administered every other day. These mice were sacrificed on day 14 to evaluate the effects of delayed intervention.

For the compound 3k-treated groups (d, e), a corresponding vehicle administration group, which received only the solvent on the same dosing schedule, was included as a control.

### Preparation and administration of test compounds

Compound 3k (MW: 345.48 g/mol) was purchased from Selleck Chemicals (Kanagawa Prefecture). It is a selective PKM2 inhibitor derived from a novel naphthoquinone compound that has demonstrated considerable biological activity. Compound 3k exhibits more potent PKM2 inhibitory activity than the traditional inhibitor shikonin and exhibits significant antiproliferative effects in several cancer cell lines (15, 26). Compound 3k was dissolved in 100 μl of a solvent mixture consisting of DMSO and corn oil (1:9 ratio) (DMSO: #D-8779, Sigma; corn oil: #25606–65, Nacalai Tesque). Compound 3k was administered to mice *via* oral gavage at doses of 1, 3, or 5 mg/kg. Mice in the vehicle control group received 100 μl of the solvent mixture without the drug.

Compound 7d (PKM2-IN-6; MW: 322.38 g/mol; CAS: 771,467–00–6) and compound 10i (PKM2-IN-3; MW: 338.40 g/mol; CAS: 2,408,841–19–8) are selective PKM2 inhibitors that have demonstrated potent biological activity in various cancer cell lines. In this present study, these compounds were only used for *in vitro* experiments and were not administered to animals.

### Cell culture

HK-2 cells (ATCC, American Type Culture Collection, Catalog Number: CRL-2190), a human kidney proximal tubular epithelial cell line, were cultured in Ham’s F-12/Dulbecco’s modified Eagle medium supplemented with 5% fetal calf serum, 100 U/ml penicillin, and 100 μg/ml streptomycin. When the cells reached confluence in 100 mm dishes, they were transferred to a serum-free medium and incubated at 37 °C for 24 h to synchronize the cell cycle.

The HK-2 cells were seeded in 12-well plates (5 × 10^4^ cells/well) for RNA extraction or 6-well plates (1 × 10^5^ cells/well) for protein extraction and incubated in a growth medium for 72 h.

### Cell injury models and compound treatment in HK-2 cells

Two *in vitro* models were employed to analyze the effects of compound 3k on human renal tubular epithelial (HK-2) cells: an OGD/R model and a TGF-β1-induced EMT model.

### OGD/R treatment

HK-2 cells were exposed to hypoxia (1% O_2_; Thermo Forma 3130 CO_2_ incubator; Thermo Fisher Scientific) and maintained in a glucose-free medium for 24 h. This condition served as the control for the OGD/R experiments. After 24 h, the cells were reoxygenated by replacing the medium with a glucose-containing medium under normal oxygen conditions (20% O_2_). During reoxygenation, 2 μM of compound 3k was added immediately upon reoxygenation and again after 48 h. Control cells received 0.1% DMSO.

EMT induction and compound 3k treatment in HK-2 cells:

HK-2 cells (ATCC, CRL-2190) were seeded into 12-well plates and cultured to confluence in Ham’s F-12/Dulbecco’s modified Eagle medium supplemented with 5% fetal calf serum, 100 U/ml penicillin, and 100 μg/ml streptomycin. Mild serum starvation was induced by incubating cells in 2.5% FCS medium for 24 h, followed by replacement with normal growth medium.

Cells were then treated for 72 h under the following conditions, with all treatments initiated at the time of medium exchange.

• Control: Vehicle (DMSO) at the same final concentration used for dissolving compound 3k.
• Recombinant human TGF-β1 (rhTGF-β1) group: 5 ng/ml recombinant human TGF-β1 (#240 B; R&D Systems).
• Compound 3k group: 2 μM compound 3k administered at 0 h and re-added at 48 h.
• TGF-β1 plus 3k group: 5 ng/ml TGF-β1 and 2 μM compound 3k added at 0 h, with compound 3k re-added at 48 h.

After 72 h of treatment, cells were collected and lysed for protein extraction. Western blotting was performed as detailed in the western blotting section.

### Cell Counting Kit-8 cell viability assay

Cell viability was assessed using CCK-8 (Dojindo) according to the manufacturer’s instructions. Briefly, HK-2 cells were seeded in a 96-well plate at a density of 5 × 10^3^ cells/well and cultured overnight to facilitate attachment. The cells were then treated with compound 3k at various concentrations (0, 2, 4, 6, 8, and 10 μM) or DMSO (vehicle control) for 48 h. After the incubation period, 10 μl of CCK-8 reagent was added to each well, and the plate was incubated at 37 °C for an additional 2 h. Absorbance of the samples was measured at 450 nm using a microplate reader (Arvo X5; PerkinElmer).

### RNA extraction and RT-qPCR

Total RNA was extracted using TRIzol (#15596018; Thermo Fisher Scientific), and cDNA synthesis was performed using the ReverTra Ace qPCR RT Kit (#FSQ-301; Toyobo Co., Ltd) according to the manufacturer’s instructions. RT-qPCR was conducted using the StepOnePlus Real-Time PCR System (Applied Biosystems, Foster City) with Thunderbird Next qPCR Mix (#QPX-201; Toyobo). Gene expression levels were normalized to those of *GAPDH*. The data are presented as the relative fold change compared to the control (control = 1). Individual data points are shown. The primer sequences used for amplification are listed in Tables S1 and S2.

### RNAi

*PKM2*-specific knockdown was performed using Lipofectamine RNAiMAX (#13778030; Thermo Fisher Scientific) for siRNA transfection, following the manufacturer’s instructions. Silencer siRNA oligonucleotides targeting the M2 isoform of pyruvate kinase (Hokkaido Science) were transfected in Opti-MEM medium (#31985062; Thermo Fisher Scientific) at a final concentration of 10 pmol. The siRNA oligonucleotide sequences used are listed in Table S2. As a negative control, cells were transfected under identical conditions with control siRNA-A (#sc-37007; Santa Cruz Biotechnology) instead of siPKM2.

### Masson’s trichrome staining

The mice were anesthetized using isoflurane (induction: 2%–3%, maintenance: 0.5%–2%) and euthanized *via* cervical dislocation under deep anesthesia. Subsequently, the left kidney tissues were harvested, fixed in 4% paraformaldehyde for 24 h, and placed in 70% ethanol. Paraffin-embedded tissue blocks were prepared, sliced into 3-μm-thick sections, and stained with Masson’s trichrome solution. Images were captured using an Olympus BX43 microscope with an Olympus DP70 camera (Olympus Corporation).

### Immunofluorescence staining

Paraffin-embedded tissue blocks were sliced into 3 μm-thick sections, as performed for Masson’s trichrome staining. The sections were deparaffinized and subjected to antigen retrieval using Immunosaver (#333; Nissin-EM) at 100 °C for 60 min. The sections were then blocked with 5% skim milk in PBS for 5 min. To detect PKM2, the sections were incubated with a primary antibody against PKM2 (rabbit anti-PKM2, 800 μg/ml, #15822-1-AP; Proteintech) diluted 1:300 in blocking buffer (5% skim milk/PBS) at 4 °C for 24 h. The primary antibody was visualized using Alexa Fluor 555-conjugated secondary antibody (goat anti-rabbit IgG, #A21428; Molecular Probes, Thermo Fisher Scientific) diluted 1:300 in blocking buffer (5% skim milk/PBS) at 20 °C–25 °C (room temperature, RT) for 1 h. Rabbit IgG, diluted 1:375 (1 mg/ml, #30000-0-AP; Proteintech), was used as a negative control. In addition, FITC-labeled LTL (#FL-1321–2, Vector) was used to stain specific kidney regions, followed by nuclear staining with DAPI (#D1306; Molecular Probes, Thermo Fisher Scientific). Finally, immunofluorescence signals were merged, and image data were acquired using a confocal laser scanning microscope (900; Carl Zeiss).

To detect YAP1 (rabbit anti-YAP1, 750 μg/ml #13584-1-AP; Proteintech) and p-β-catenin (Ser552: rabbit anti-p-β-catenin, 50 μg/ml #4176, CST; Ser675: rabbit anti-p-β-catenin, 310 μg/ml #5651, CST), tissue sections were incubated with primary antibodies diluted in blocking buffer (1% BSA/PBS) at 4 °C for 24 h (YAP1: 1:300; p-β-catenin: 1:100). The primary antibodies were visualized with Alexa Fluor 555-conjugated secondary antibodies (goat anti-rabbit IgG, #A21428; Molecular Probes, Thermo Fisher Scientific) diluted in blocking buffer (1% BSA/PBS) at RT for 1 h. Rabbit IgG, diluted 1:500 (1 mg/ml, #30000-0-AP, Proteintech), was used as a negative control. FITC-labeled LTL was used to stain the kidney region, followed by DAPI nuclear staining. Immunofluorescence signals were then merged. Data were acquired using a confocal laser scanning microscope (900; Carl Zeiss).

### Western blotting

Proteins were extracted from HK-2 cells using RIPA buffer supplemented with a protease inhibitor cocktail for mammalian cell and tissue extracts (#25955–11; Nacalai Tesque) and a phosphatase inhibitor cocktail (#07574–61; Nacalai Tesque). Lysates were homogenized using a sonicator. Protein concentrations were determined using a BCA Protein Assay Kit (#06385; Nacalai Tesque).

Samples were prepared by mixing with 4 × Laemmli buffer and heating at 100 °C for 5 min. Equal amounts of protein (15 μg) were subjected to 12% SDS-PAGE at 200 V for 60 min. The proteins were transferred onto polyvinylidene fluoride membranes at 180 mA for 65 min. The membranes were then blocked with 5% BSA and incubated with primary antibodies overnight at 4 °C.

Primary antibodies against the following proteins were used: CCN2 (CTGF) (D8Z8U) (#86641; CST), phosphorylated YAP (Tyr357) (#ab62751; Abcam), YAP1 (#13584-1-AP; Proteintech), PKM2 (#15822-1-AP; Proteintech), Pan-TEAD (D3F7L) (#13295; CST), PKM1 (#15821-1-AP; Proteintech), β-catenin (D10A8) (#8480; CST), lamin B1 (#PM064MS; MBL), GAPDH (D16H11) (#5174; CST), Smad2/3 (D7G7) (#8685; CST), Phospho-Smad3 (Ser423/425) (#GT1207; GTX), and TEAD2 (#21159-1-AP; Proteintech). Goat anti-rabbit IgG (#P04448; Dako) were used as the secondary antibody. Protein signals were detected using enhanced chemiluminescence (ECL Prime Western Blotting Detection Reagent, RPN2232; GE Healthcare). MW standards were visualized using Protein Ladder One Plus, Tri-color for SDS-PAGE (#19593–25; Nacalai Tesque). Band intensity was quantified using the ImageQuant LAS 4000 system (GE Healthcare; https://rega.kuleuven.be/bac/economou/files/pdf/LAS4000/imagequant-las4000-biomolecular-imager.pdf).

### Western blotting of cytoplasmic and nuclear proteins

To analyze protein localization, fractionated proteins were extracted from HK-2 cells. The cells were pelleted and washed twice with PBS; the final pellet was resuspended in 100 μl of buffer A (10 mM Hepes, pH 7.9; 10 mM KCl; 0.1 mM EDTA; 0.1 mM EGTA; 1 mM DTT; 0.5 mM PMSF; 1% protease inhibitor). The suspension was incubated on ice for 15 min, and nuclear protein (NP)-40 was added to a final concentration of 10%. The samples were vortexed for 10 s and centrifuged at 500×*g* for 3 min. The supernatant containing the cytoplasmic proteins was collected, and the pellet was washed once with 100 μl of buffer A. The sample was subsequently centrifuged again at 500×*g* for 1 min, and the supernatant was discarded.

The nuclear pellet was resuspended in 50 μl of buffer B (20 mM Hepes, pH 7.9; 0.4 M NaCl; 1 mM EDTA; 1 mM EGTA; 1 mM DTT; 0.5 mM PMSF; 1% protease inhibitor) and incubated on ice for 15 min. The samples were centrifuged at 204×*g* for 15 min, and the resulting supernatant containing the NPs was collected (63). Protein concentrations were determined using a BCA Protein Assay Reductant-Compatible Kit (Nacalai Tesque).

For Western blot analysis, 10 μg of either cytoplasmic protein or NP was loaded, following the same protocol used for the total protein analysis.

### Cross-linking PKM2 to assess tetramer, dimer, and monomer formation

For the cross-linking assay in HK-2 cells, proteins were prepared using disuccinimidyl suberate (DSS; Abcam) according to the manufacturer’s instructions. DSS was added to a final concentration of 1 mM to cross-link proteins at RT for 30 min. The reaction was quenched with 20 mM Tris for 15 min. The cross-linked samples were prepared by mixing 30 μg of protein with 4 × Bolt lithium dodecyl sulfate sample buffer (Invitrogen), followed by boiling for 5 min. The proteins were then separated using 12% SDS-PAGE for analysis.

### Co-IP

HK-2 cells were subjected to OGD/R treatment and subsequently lysed in RIPA buffer supplemented with 1% protease inhibitor cocktail (#25955–11; Nacalai Tesque) and 1% phosphatase inhibitor cocktail (#07574–61; Nacalai Tesque). Co-IP was performed using 25 μg of Dynabeads M-280 sheep anti-rabbit IgG (Veritas), according to the manufacturer’s instructions. Briefly, the cell lysates were incubated with 2.4 μg of anti-PKM2 antibody (Proteintech #15822-1-AP) overnight at 4 °C under gentle rotation. The antibody–protein complexes were captured using Dynabeads M-280 sheep anti-rabbit IgG. The immunocomplexes were eluted with 4 × Laemmli sample buffer. The eluted proteins were analyzed using western blotting with antibodies against YAP1 and β-catenin. Rabbit serum was used as a control for nonspecific immunoprecipitation. In addition, an aliquot of total protein lysate ("input") was loaded as an input control to confirm the presence of YAP1 and β-catenin in the starting material.

### Statistical analysis

Statistical analyses were performed using JMP Pro 17.0.0 software (SAS Institute Inc., Cary; https://www.jmp.com/en/software/predictive-analytics-software). The specific statistical methods applied are detailed in the respective figure legends. All experiments were independently performed at least twice, each in duplicate or triplicate. Given the non-normal distribution of the data, the results are expressed as the median with IQR. Group differences were evaluated using Steel’s test, with statistical significance set at *p* < 0.05 compared to the vehicle group. Additionally, the Wilcoxon signed-rank test was employed for paired comparisons. A *p*-value less than 0.05 were considered significant. Individual data points are shown as the median and IQR.

### Study approval

All animal experiments were approved by the Institutional Animal Care and Use Committee of Saitama Medical University (Protocol no. 3678), and conducted in accordance with the ARRIVE 2.0 guidelines to ensure comprehensive and transparent reporting of the study design, methodology, and analysis.

## Data availability

Supporting data values for all graphs and data presented as medians (IQR) are provided in a separate XLS file submitted as a Supporting Information file. Original, unedited gel and blot images are also provided as Supporting Information Figures.

## Supporting information

This article contains supporting information.

## Conflict of interests

The authors declare that they have no conflicts of interest with the contents of this article.
