# Supplementary material for: PKM2 inhibitor suppresses kidney fibrogenesis by disrupting YAP-TEAD-CCN2 transcriptional signaling following ischemia–reperfusion injury
Source: J Biol Chem. 2025 Dec 9;302(1):111029. doi: 10.1016/j.jbc.2025.111029 (PMC12803839; doi:10.1016/j.jbc.2025.111029)
Supplement: Supplementary Material 1 [file mmc1.pdf]

SUPPORTING INFORMATION

PKM2 inhibitor suppresses kidney fibrogenesis by disrupting YAP-TEAD-CCN2 transcriptional signaling following ischemia–reperfusion injury

Wakako Kosakai[1], Tsutomu Inoue[1], Tetsuya Sato[2], Hirokazu Okada[1]*

[1] Department of Nephrology, Faculty of Medicine, Saitama Medical University, Saitama, Japan

[2] Biomedical Research Center, Faculty of Medicine, Saitama Medical University, Saitama, Japan

List of contents:

Supporting Figures S1-7, Unedited gel and blot images, and Supporting Tables S1 and S2

S. Figure 1

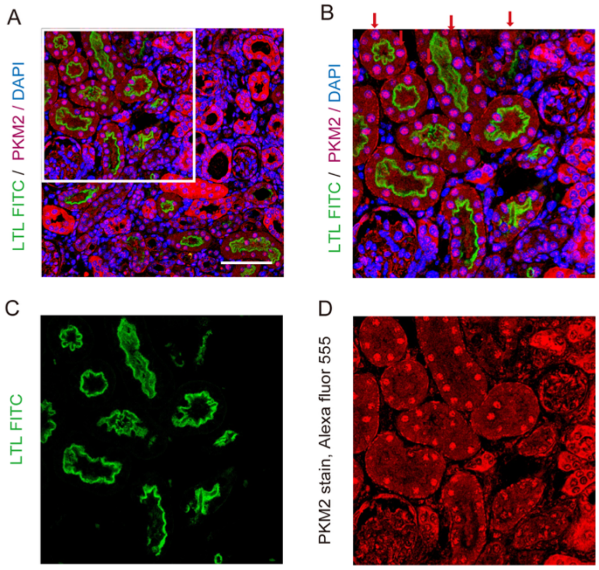

Supporting Figure S1. Nuclear localization of PKM2 in the mouse kidney cortex 14 days after unilateral ischemia–reperfusion injury (UIRI).

(A) Representative image of a kidney cortical section stained for PKM2 (Alexa Fluor 555; red), *Lotus tetragonolobus* lectin (LTL, FITC; green), and DAPI (blue; nuclei). The inset indicates the region shown at higher magnification in panel B. Scale bar: 100 µm.

(B) Magnified, merged image of the region outlined in panel A. Red arrows indicate nuclear localization of PKM2.

(C) LTL staining (FITC, green channel only) for proximal tubular structures in the region shown in panel B.

(D) PKM2 staining (Alexa Fluor 555, red channel only) in the same region.

DAPI was used as a nuclear counterstain and is shown in the merged images. Images were acquired using a confocal laser scanning microscope. No scale bars are included in panels B–D.（The LTL FITC, LTL FITC/PKM2/DAPI, and PKM2 panels of the Day 14 vehicle-treated IRI group column in Figure 3B correspond to the same microscopic field as Supporting Figure S1A–D, and the exact same images are digitally enlarged in Supporting Figure S1 to more clearly illustrate PKM2 subcellular localization.）

S. Figure 2

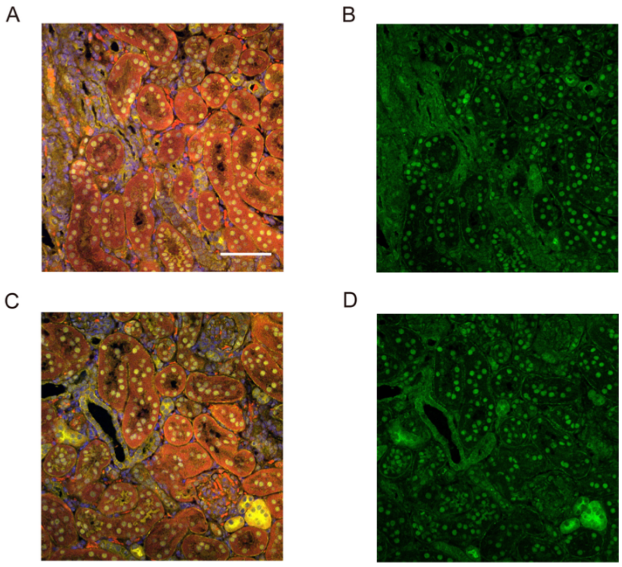

Supporting Figure S2. Cortical proximal tubules in γGT-Cre × tdTomato F1 mice following UIRI (14 days), visualized by fluorescence staining (paraffin sections).

Representative confocal images are shown:

- **Left panels (A, C):** Merged images showing tdTomato (red; γGT-Cre-positive proximal tubules), Alexa Fluor 488 conjugated anti-PKM2 antibody (green; PKM2), and DAPI (blue; nuclei). In the merged images, tdTomato-positive tubules appear orange, and the overlap of PKM2 and DAPI within nuclei produces white to yellow coloration, indicating nuclear PKM2 expression.
- **Right panels (B, D):** Alexa Fluor 488 signal (PKM2, green channel only) in the same fields shown in the merged images, highlighting PKM2 localization within the cortex.

All images were obtained from kidney sections at day 14 post-UIRI. Scale bars: 100 µm.

S. Figure 3

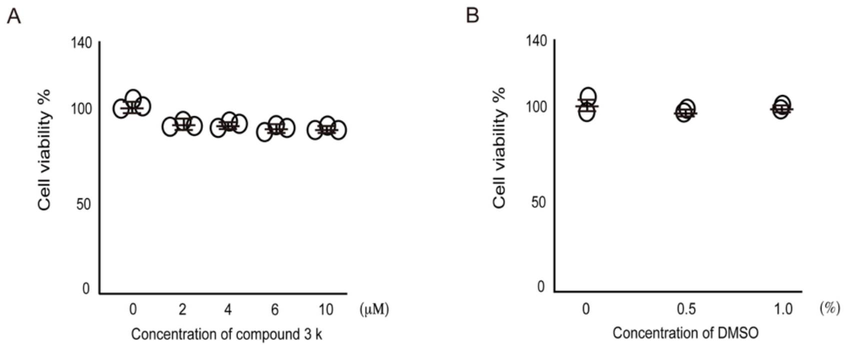

Supporting Figure S3. Viability of HK-2 cells determined using the CCK-8 assay.

(A) Effect of compound 3k (0–10 μM) on HK-2 cell viability.

(B) Effect of DMSO (0–1%) on HK-2 cell viability.

HK-2 cells were treated with the indicated concentrations of compound 3k or DMSO for 24 h. Cell viability was assessed using the CCK-8 assay, and results are expressed as the median with interquartile range (IQR). Statistical comparisons were performed using Steel's test, with $P < 0.05$ considered significant; for panel A each concentration group was compared to the 0 μM (control) group, and for panel B each DMSO concentration was compared with the 0% (control) group. Individual data points represent the median and IQR. See the Materials and Methods section for further details.

S. Figure 4

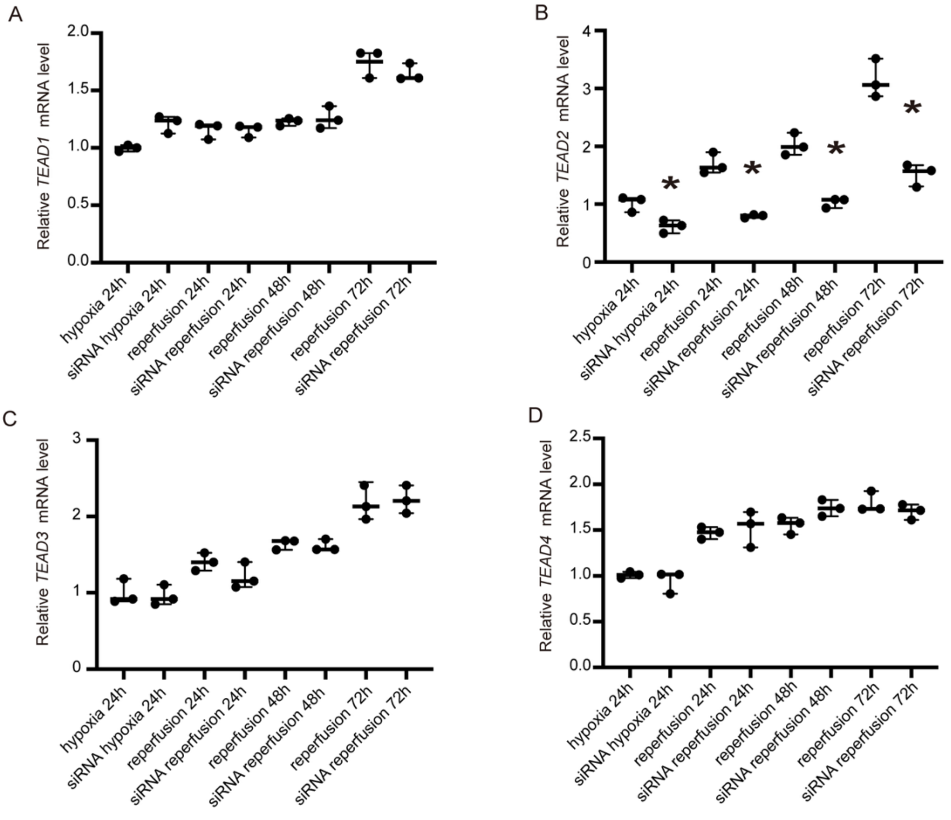

Supporting Figure S4. RT-qPCR analysis of TEAD family expression in HK-2 cells following PKM2 knockdown.

(A–D) Relative mRNA expression levels of TEAD1 (A), TEAD2 (B), TEAD3 (C), and TEAD4 (D) in HK-2 cells transfected with siRNA targeting PKM2 (siPKM2) or control siRNA, as determined by RT-qPCR.

First, cells were transfected with siPKM2 or control siRNA and subsequently subjected to OGD/R treatment as described in the protocol for Figure 7A. Total RNA was then extracted and cDNA synthesized as described in the Materials and Methods section. Gene expression was normalized to GAPDH and expressed as fold change relative to the control group (control = 1). Individual data points are shown. Statistical analysis was performed as described in the Materials and Methods section. The primer and siRNA sequences are listed in Tables S1 and S2.

S. Figure 5

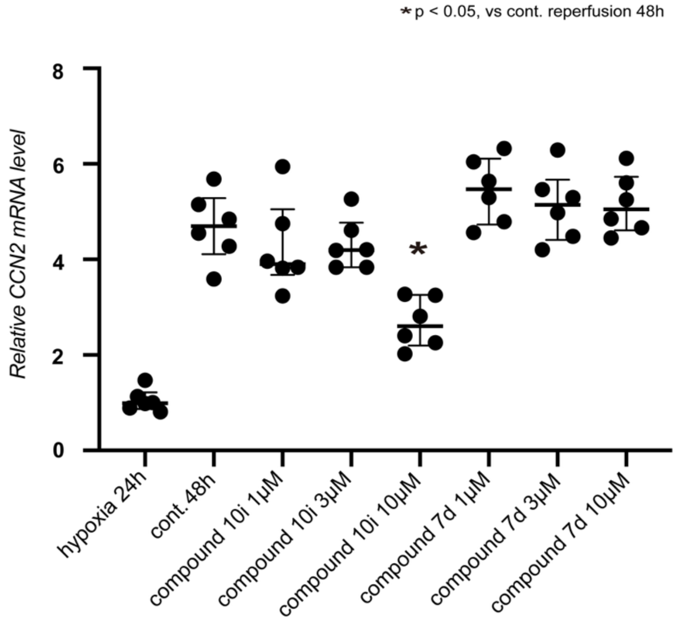

Supporting Figure S5. Effects of compounds 10i and 7d on CCN2 mRNA expression in OGD/R-injured HK-2 cells.

HK-2 cells were subjected to OGD/R to induce cell injury and subsequently treated with compounds 10i or 7d at concentrations of 1, 3, or 10 μM. CCN2 mRNA levels were measured by RT-qPCR 48 h after reoxygenation. Expression levels at 24 h after OGD (control) were normalized to 1. Statistical comparisons between each treatment group and the control group (reoxygenation 48 h) were performed using Steel's test. * indicates statistical significance ($P < 0.05$). Individual data points represent the median and interquartile range (IQR) (N=6).

S. Figure 6

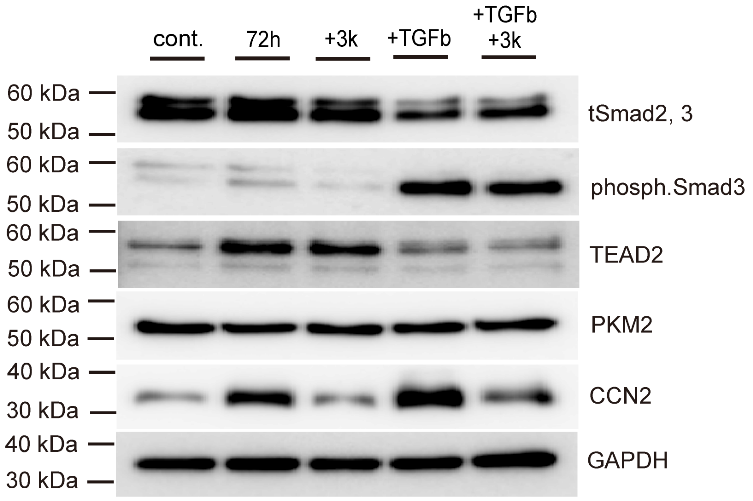

Supporting Figure S6. Western blot analysis of EMT- and fibrosis-related proteins in HK-2 cells treated with TGF-β and/or compound 3k.

HK-2 cells were cultured in 2.5% FCS medium for 24 h, then returned to normal medium and treated for 72 h under the indicated conditions: control (cont.), compound 3k (2 μM), TGF-β (5 ng/mL), or TGF-β + 3k (2 μM). Compound 3k was administered again at 48 h. At 72 h, proteins were extracted and subjected to western blotting. Blots were probed for tSmad2/3, phospho-Smad3, TEAD2, PKM2, CCN2, and GAPDH (loading control). A representative result from three independent experiments (N = 3) is shown.

S. Figure 7

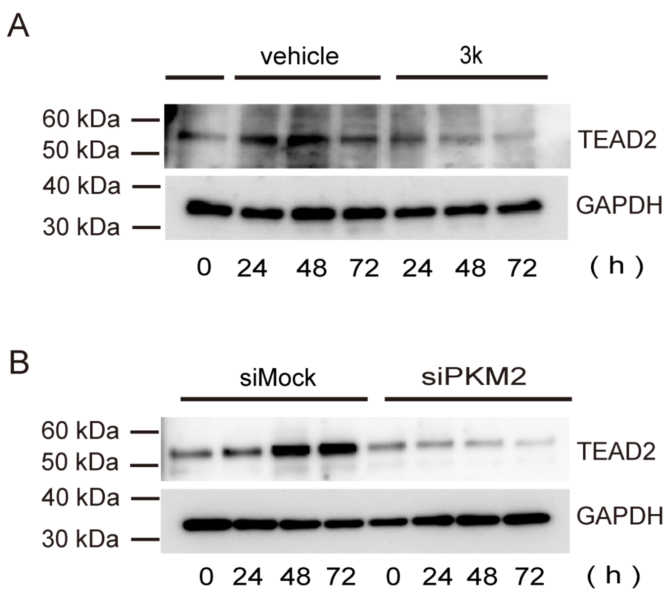

Supporting Figure S7. Western blot analysis of TEAD2 expression in HK-2 cells subjected to OGD/R and PKM2 inhibition or knockdown.

(A) TEAD2 protein expression after treatment with compound 3k (2 µM, administered at the onset of reoxygenation and again at 48 h) or vehicle (DMSO) under OGD/R conditions.

(B) TEAD2 protein expression in HK-2 cells transfected with siPKM2 or mock siRNA under OGD/R conditions.

Protein lysates were collected at the indicated time points (0, 24, 48, and 72 h) following reoxygenation. Western blotting was performed using a TEAD2-specific antibody (Proteintech #21159-1-AP) according to the protocol described in the "Western blotting" section in the Experimental procedures. GAPDH served as a loading control.

The blots shown are representative of six independent experiments (N=6). (The GAPDH loading control for panel A is identical to that in Figure 8B, as both panels were derived from the same membrane and exposure that was reprobed for TEAD2.)

Full unedited gels for Figure8A

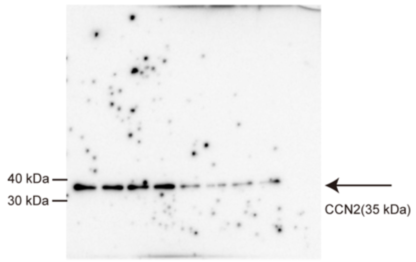

CTGF antibody (D8Z8U, Cell Signaling)

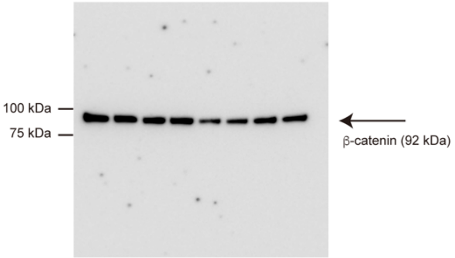

β-catenin antibody (D10A8, Cell Signaling)

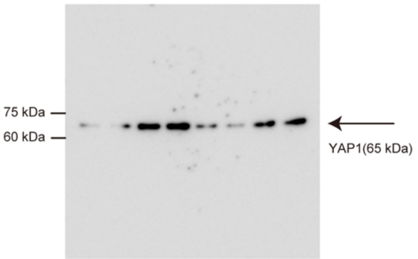

YAP1 antibody (#13584-1-AP,proteintech)

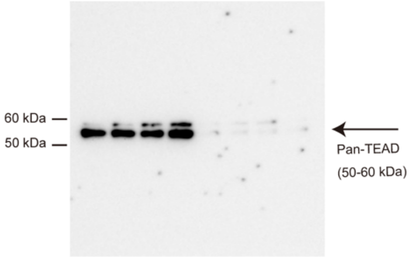

Pan-TEAD antibody (D3F7L, Cell Signaling)

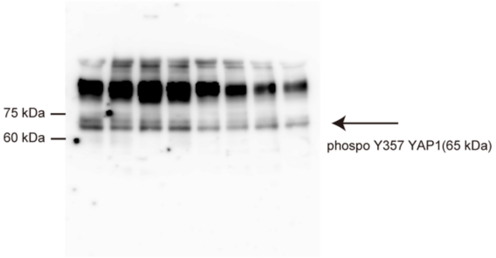

phospo Y357 YAP1 antibody (#ab62751,abcom)

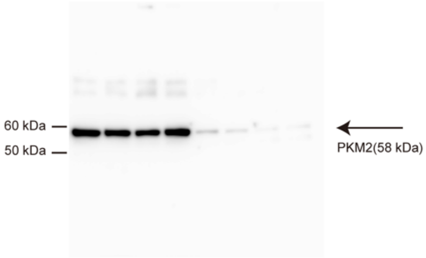

PKM2 antibody (#15822-1-AP,proteintech)

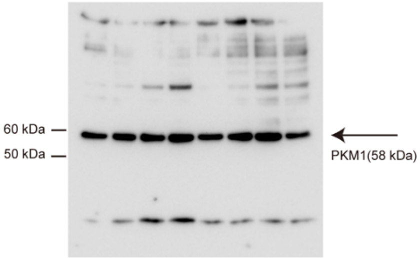

PKM1 antibody (#15821-1-AP,proteintech)

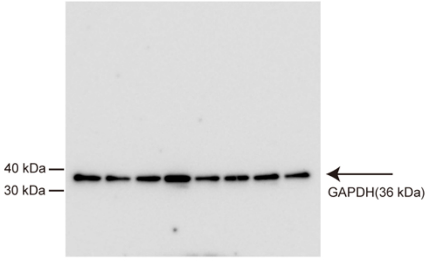

GAPDH antibody (D16H11,Cell Signaling)

Full unedited gels for Figure 8B

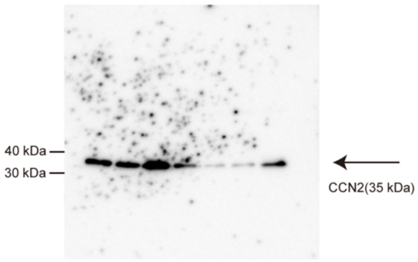

CTGF antibody (D8Z8U, Cell Signaling)

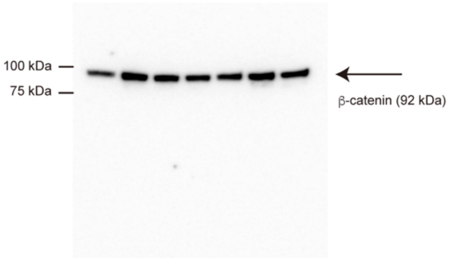

β-catenin antibody (D10A8, Cell Signaling)

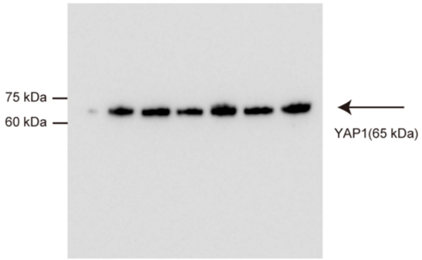

YAP1 antibody (#13584-1-AP,proteintech)

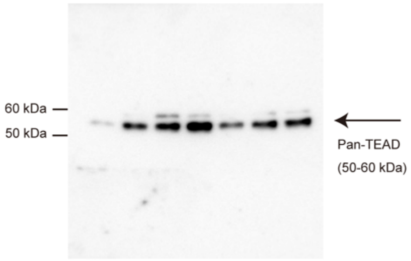

Pan-TEAD antibody (D3F7L, Cell Signaling)

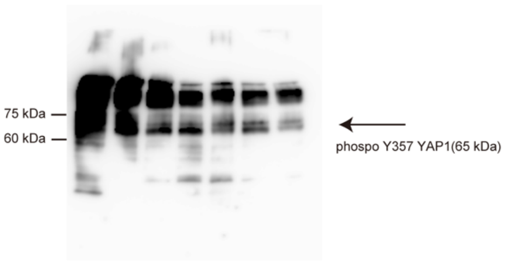

phospo Y357 YAP1 antibody (#ab62751,abcom)

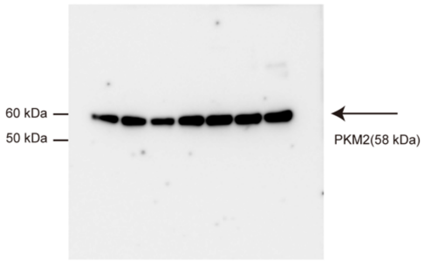

PKM2 antibody (#15822-1-AP,proteintech)

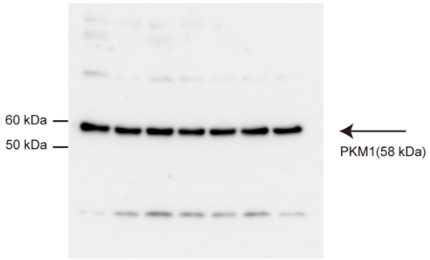

PKM1 antibody (#15821-1-AP,proteintech)

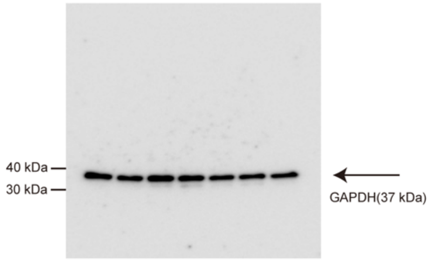

GAPDH antibody (D16H11,Cell Signaling)

Full unedited gels for Figure 9A

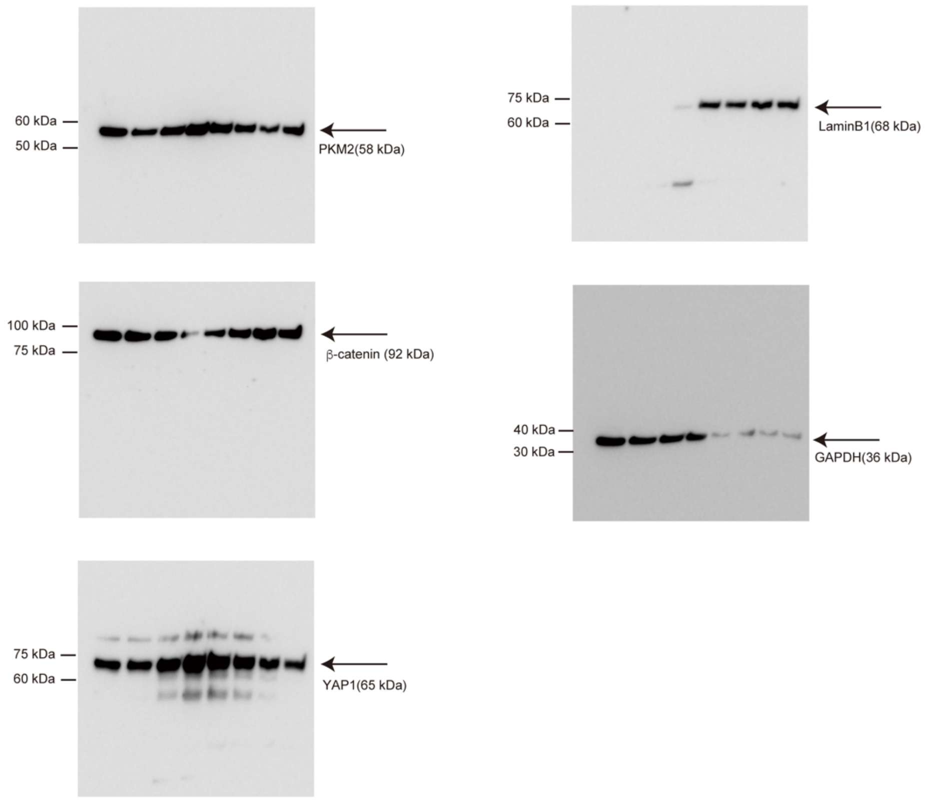

Full unedited gels for Figure 9B

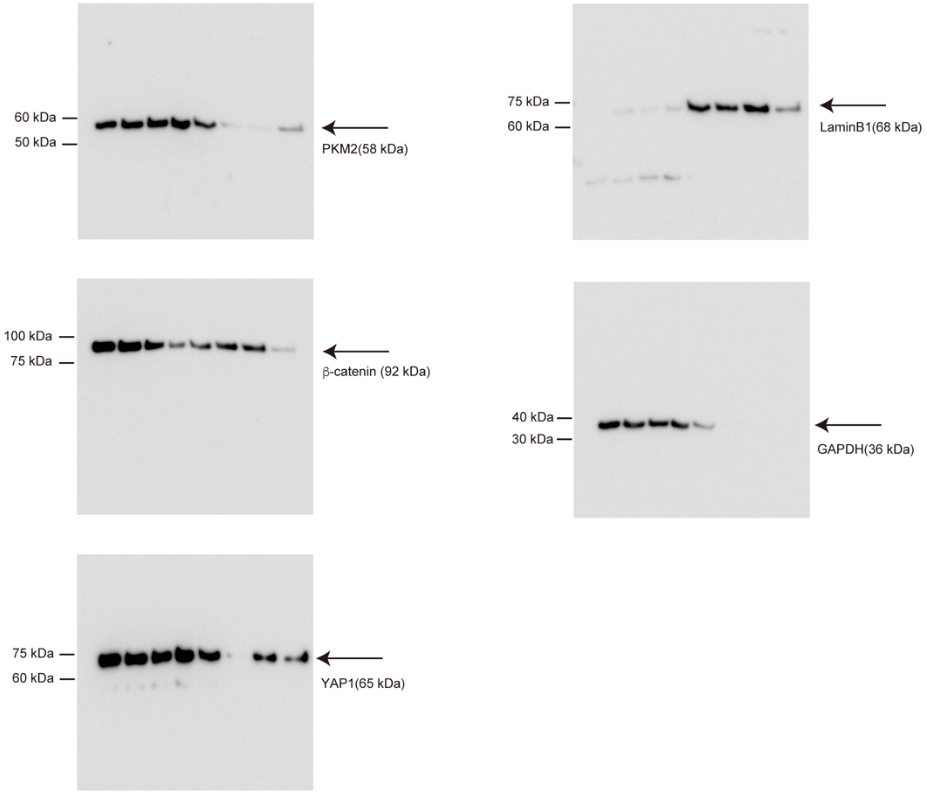

Full unedited gels for Figure 9C

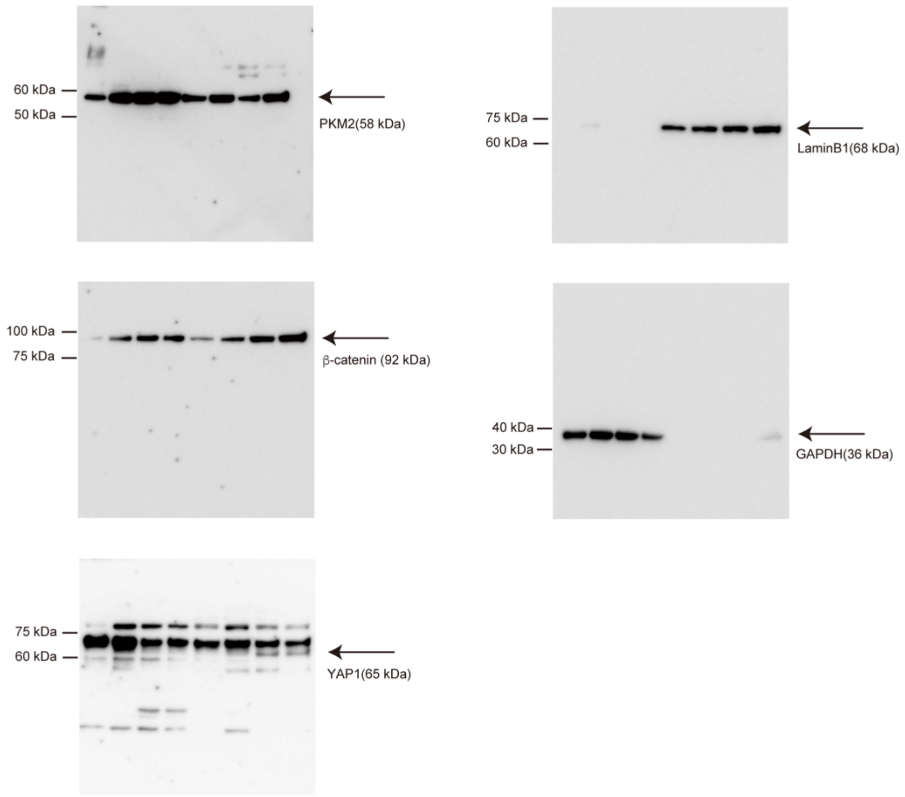

Full unedited gels for Figure 9D

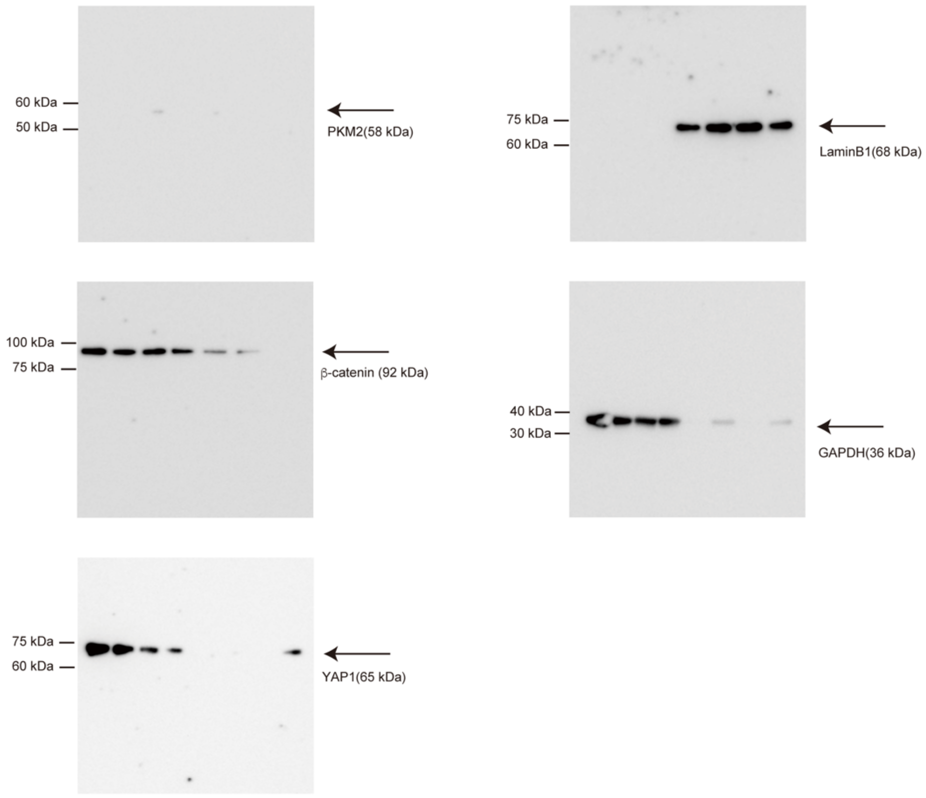

Full unedited gels for Figure S6

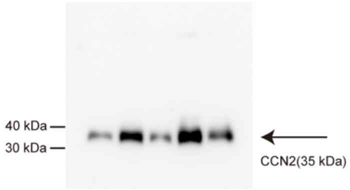

CTGF antibody (D8Z8U, Cell Signaling)

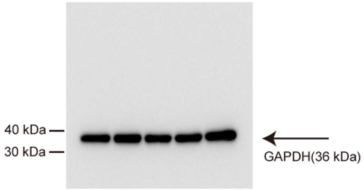

GAPDH antibody (D16H11,Cell Signaling)

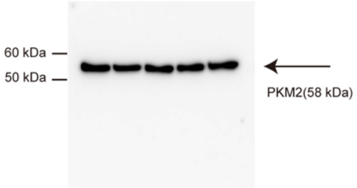

PKM2 antibody (#15822-1-AP,proteintech)

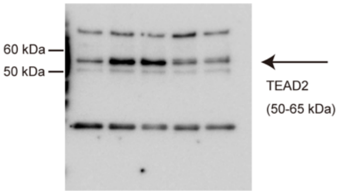

TEAD2 antibody (#21159-1-AP,proteintech)

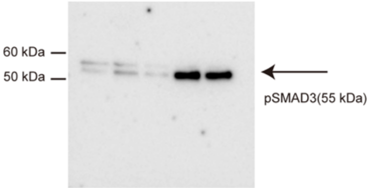

Phospho-Smad3 Ser423/425 antibody (#GT1207,GTX)

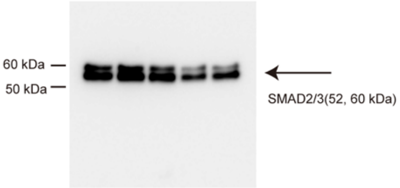

Smad2/3 antibody (D7G7,Cell Signaling)

Full unedited gels for Figure S7A

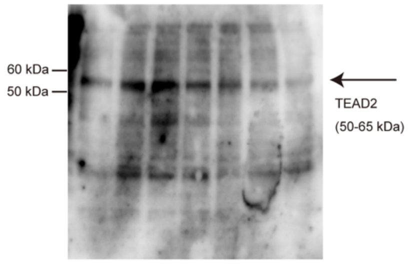

TEAD2 antibody (#21159-1-AP,proteintech)

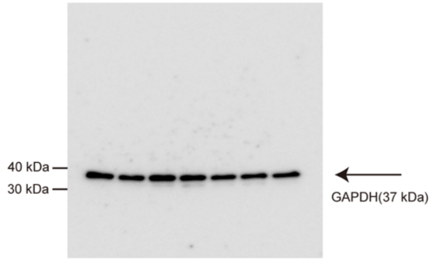

GAPDH antibody (D16H11,Cell Signaling)

Full unedited gels for Figure S7B

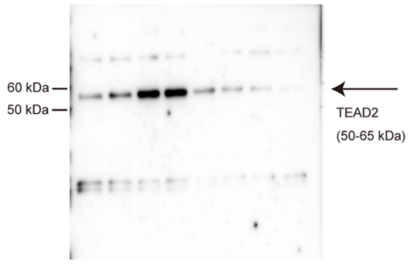

TEAD2 antibody (#21159-1-AP,proteintech)

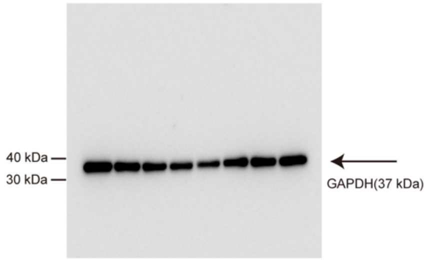

GAPDH antibody (D16H11,Cell Signaling)

Gene Name	Symbol	FW Sequence (5' → 3')	RV Sequence (5' → 3')	Species
Collagen Type1 alpha	*Col1a*	AGACATGTTCAGCTTTGTGGAC	GCAGCTGACTTCAGGGATG	mouse
Fibronectin EIIIA Isoform	*Fn-EIIIA*	ATCCGGGAGCTTTTCCCTG	TGCAAGGCAACCACACTGAC	mouse
CCN2	*Ccn2*	GGGCCTCTTCTGCGATTTC	ATCCAGGCAAGTGCATTGGTA	mouse
	CCN2	AATGCTGCGAGGAGTGGGT	CGGCTCTAATCATAGTTGGGTCT	human
Pyruvate kinase M 2	*Pkm2*	CAGAGAAGGTCTTCCTGGCTCA	GCCACATCACTGCCTTCAGCAC	mouse
	PKM2	ATGGCTGACACATTCCTGGAGC	CCTTCAACGTCTCCACTGATCG	human
Yes associated protein 1	*Yap1*	ACCCTCGTTTTGCCATGAAC	TGTGCTGGGATTGATATTCCGTA	mouse
	YAP1	TAGCCCTGCGTAGCCAGTTA	TCATGCTTAGTCCACTGTCTGT	human
lyceraldehyde 3-phosphate dehydrogenas	*Gapdh*	CTCCCACTCTTCCACCTTCG	GCCTCTCTTGCTCAGTGTCC	mouse
	GAPDH	CTCTGCTCCTCCTGTTCGAC	ACGACCAAATCCGTTGACTC	human
TEA domain transcription factor 1	*Tead1*	GGCCGGGAATGATTCAAACAG	CAATGGAGCGACCTTGCCA	mouse
	TEAD1	AAGCTGAAGGTAACAAGCATGG	GCTGACGTAGGCTCAAACCC	human
TEA domain transcription factor 2	*Tead2*	GAAGACGAGAACGCGAAAGC	GATGAGCTGTGCCGAAGACA	mouse
	TEAD2	CCTTCTCTGCAGGCCAAA	GCTCGTACCCTGGGAGGT	human
TEA domain transcription factor 3	*Tead3*	CGACAGCATGACCATCAGC	GTCCCGACTTGTGACCACC	mouse
	TEAD3	GCCGGCGGAAGATCATCCTG	TCGTCCGAGTCTTCCCCGTC	human
TEA domain transcription factor 4	*Tead4*	CAACCTGGAACATCCCACGAT	GAAAGCCGAGAACTCCAACAT	mouse
	TEAD4	GCCCGCTACATCAAGCTCCG	CGAGCCAGCACCTGGATGTG	human

Supporting Table S1. Primer sequences used for RT-qPCR

siRNA				
si Pyruvate kinase M 2	*siPKM2*	CCAUAAUCGUCCUCACCAATT	UUGGUGAGGACGAUUAUGGTT	human
Control siRNA-A:sc-37007	*Control siRNA*	No sequence information	No sequence information	human

Supporting Table S2. siRNA sequences used for gene silencing experiments
